# Influences of Forest Structure, Climate and Species Composition on Tree Mortality across the Eastern US

**DOI:** 10.1371/journal.pone.0013212

**Published:** 2010-10-13

**Authors:** Emily R. Lines, David A. Coomes, Drew W. Purves

**Affiliations:** 1 Forest Conservation and Ecology Group, Department of Plant Sciences, University of Cambridge, Cambridge, United Kingdom; 2 Microsoft Research Cambridge, Cambridge, United Kingdom; University of Zurich, Switzerland

## Abstract

Few studies have quantified regional variation in tree mortality, or explored whether species compositional changes or within-species variation are responsible for regional patterns, despite the fact that mortality has direct effects on the dynamics of woody biomass, species composition, stand structure, wood production and forest response to climate change. Using Bayesian analysis of over 430,000 tree records from a large eastern US forest database we characterised tree mortality as a function of climate, soils, species and size (stem diameter). We found (1) mortality is U-shaped vs. stem diameter for all 21 species examined; (2) mortality is hump-shaped vs. plot basal area for most species; (3) geographical variation in mortality is substantial, and correlated with several environmental factors; and (4) individual species vary substantially from the combined average in the nature and magnitude of their mortality responses to environmental variation. Regional variation in mortality is therefore the product of variation in species composition combined with highly varied mortality-environment correlations within species. The results imply that variation in mortality is a crucial part of variation in the forest carbon cycle, such that including this variation in models of the global carbon cycle could significantly narrow uncertainty in climate change predictions.

## Introduction

An understanding of tree mortality is central to any predictive understanding of forest dynamics. The long-term dynamics of woody biomass are regulated by the difference between gains through individual growth and losses through mortality. This makes tree mortality a crucial determinant of the forest carbon cycle, the future of which is a major source of uncertainty in Earth System Model predictions of future climate [1]. Moreover, differences in mortality rates among species appear to be major determinants of ecological succession [2], [3], the geographical ranges of species [4], [5], stand structure (e.g. stem size distributions: [6], [7]), and responses of forests to climate change and disease [8], [9]. However, we currently have little quantitative information about the nature, magnitude or causes of geographical variation in tree mortality.

The simplest approach to making predictions about mortality in a changing world would be to correlate stand-level mortality obtained from permanent plot data with climatic variables, and use these relationships to predict changes under future climate scenarios. The problem with this approach is that it neglects the effects of species, individual size and competition, factors that individually have been shown to strongly affect mortality at the scale of the individual tree, with potentially serious consequences for landscape-level predictions. In order to predict the impacts of changing climate on forest-level mortality, it is therefore important to isolate the effects of these factors because they are likely to show complex, semi-independent changes in the future. For example, in much of the temperate zone, many forest stands are successional and regenerating, undergoing directional change in species composition independent of any changes in the environment [10], [11]. Additionally, species are unlikely to disperse rapidly enough to track their optimal climatic conditions under rapid anthropogenic climate change, leading to combinations of species composition and environment that do not occur currently [12], [13]. Tree-level mortality patterns can also be confounded by external actions: harvesting can create various novel combinations of basal area, size distributions and species composition (e.g. [14], [15]), and pests and pathogens are often highly species-specific (e.g. sudden oak death: [16], [17]). To estimate the individual effects of each factor, it is necessary to study factors simultaneously, in order to tease apart their individual effects, otherwise the apparent effect of one is likely to be confounded by the others (e.g. apparent differences in species' average mortality rates might reflect differences in the average environments occupied by those species: [18]).

Here we use the Eastern USA Forest Inventory and Analysis (FIA) dataset to parameterise, for each of 21 common US tree species, a logistic regression model that assigns an annual probability of mortality to an individual tree given its size, species identity, competitive environment (plot basal area) and physical environment. We estimate the nature and relative magnitude of the different factors affecting tree mortality and parameterise a model that could be useful in predicting potential responses of US forest carbon stocks to climate change (e.g. [19]). Here we report: (1) how each factor affects the mortality rate of individual trees; (2) whether, and how, species differ in their underlying mortality rates and responses to size, competition and the environment; and (3) differences in the environmental dependency of forest stand-level vs. species-level mortality, which determine the level of model complexity required to accurately predict forest mortality in a changing environment.

## Materials and Methods

### Forest Inventory data

We used the pre-1999 USA Department of Agriculture Forest Inventory and Analysis (USDA FIA) dataset containing tree-level data for 182 species from a network of plots distributed across the Eastern USA [20]. The data comes from forest inventory plots which were surveyed in the 1980s and again in the 1990s, although the interval between surveys differs between states between 1 and 21 years (93% of survey intervals were between 6 and 15 years). Surveys were taken using a two-phase sampling procedure known as double sampling for stratification. In the first phase random points were chosen on aerial photographs and classified by land cover and forest type, and in the second a random subsample of each class were selected and established as field plots. Five or more points were chosen within each plot, around which several sub-plots were established and sampled using variable radius sampling, whereby the effective subplot size differs according to tree size (for more details see [21]). Species, size (diameter breast height, dbh) and status (alive, dead from harvesting, dead from natural causes) were recorded for each tree sampled, along with plot basal area (m^2^ ha^−1^). The FIA survey was designed specifically to allow accurate estimates of average forest characteristics such as species composition and average tree size through scaling from the tree, through the stand, to the regional level [20], [21].

Before analysis began, the dataset was filtered to include only those dead trees that we could be certain were not removed by human activity, and to remove various kinds of errors in the data (e.g. false mortality events corresponding to subplots that were measured in the first, but not the second, survey). The model was parameterised for 21 of the most common species, using 438,401 individual tree records in total, accounting for around 60% of all trees in the reduced dataset. Due to the high number of possible predictors being considered, only species with over 10,000 individuals in the data set were used for parameterising the model. Of these, two species (*Ulmus americana* and *Abies balsamea*) were known to have suffered severely from disease and pests during the survey period. Other species are likely also to suffer a variety of impacts from diseases which are part of the mortality patterns studied here. However, the disease impacts on *Ulmus americana* and *Abies balsamea* are known to be so severe, episodic, and localised, that in our opinion it was better to exclude both species from the analysis. Since these factors were not included as predictors of mortality in our model we did not include these species in the model fitting. We also did not consider the effects of other disturbances, both natural (e.g. fire and hurricanes) and human, on the observed mortality in the dataset. Such disturbances are likely to have had an marked effect on current species composition [22]–[24] and demographic rates [25], but are likely to be complex and interacting and, combined with a lack of a detailed land-use history, the quantification of such disturbances and evaluation of their effects may be unachievable in many areas [26].

### Environmental data

Since little is known about the geographical variation in tree mortality we had little information to judge which climatic factors might correlate with mortality. However, there have been many studies linking growth with a wide variety of climatic variables; for example, solar radiation, [27], [28], precipitation and drought [29]–[31], temperature [32], severe frost [33] and wind speed [34]. Since many studies link individual rates of mortality within a species as a function of growth (e.g. [2], [35]–[37]) there is reason to believe that mortality also varies with many different climatic variables. Our approach was therefore to assess which of these variables were most closely correlated with observed mortality patterns, rather than to attempt to generate hypotheses, in order to determine which were most important within our data.

We assigned environmental factors to each tree using two sources of environmental data, both available on a 0.5°×0.5° degree. The first source was the CRU05 climatology product (Climatic Research Unit, University of East Anglia: [38]) which provides monthly averages for many climate variables including temperature, precipitation, frost frequency, vapour pressure, cloud cover and wind speed (monthly average refers to the average over the period 1961–1990). We took the mean of each climate variable rather than climate observed over the survey period associated with each tree (which differs from tree to tree). From the CRU05 data we calculated the additional metrics of minimum temperature, degree days and average warm season (as opposed to annual) precipitation. The second source of environmental data was the Vegetation/Ecosystem Modelling and Analysis Project (VEMAP) [39], a multi-institutional project to develop a database of climate, soils and vegetation on a 0.5° latitude/longitude grid across the United States for use with ecosystem physiology models. From this source, we took only the data on US soil, which included over 20 different metrics including soil depth, and measures of soil texture. In addition the FIA provided data on soil texture for each inventory plot, divided into five classifications from xeric (normally low or deficient in available moisture), through mesic (normally moderate but adequate available moisture) to hydric (normally abundant or overabundant moisture all year) [40]. The classification of each FIA site into one of these five soil classes is intended to be independent of the climate (e.g. rainfall) at that site.

To avoid convergence problems during parameter estimation, we applied principal component analysis (PCA) to the 14 different environment variables (both from the VEMAP and CRU05 data) to remove highly correlated variables. Among highly correlated variables, the variable with the highest weighting in the principal components was retained and the rest discarded. This left four CRU05-derived climatic variables (radiation, yearly precipitation, mean annual temperature and maximum wind speed) to be included as possible mortality predictors, plus one FIA soil texture classification associated with each tree. We normalised each factor (i.e. subtracted the mean value and divided by the standard deviation) to allow for a simple comparison between the magnitudes of effects of each of the factors. We also check that plot basal area was not highly correlated with the remaining climate variables.

### Model description

Tree mortality is a difficult property to estimate because unlike growth, it has only 2 possible outcomes from each re-measured tree (survived or died), and typical tree mortality rates are low (on the order of 0.1 to 2% year^−1^), such that large sample sizes and/or long re-measurement periods are required. Moreover this dataset contained varying re-measurement intervals, meaning that a simple ‘proportion dead’ would not have been informative [3]. We therefore chose to parameterize a model describing the annual probability of death for each individual tree *i*, P(mortality, *i*). Since P(mortality, *i*) must lie between 0 and 1, we used a logistic transformation
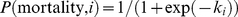
(1)where *k_i_* (which can vary from ± ∞) is a function of the predictor variables.

We included different combinations of the predictor variables: dbh (continuous); soil type (discrete, ranging from 1–5); plot basal area (i.e. FIA inventory plot) (continuous); and environmental variables (all continuous) as follows:
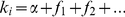
(2)where α is a constant parameter, and *f*_1_ is a function of the first predictor variable (e.g. dbh), *f*_2_ is a function of the second (e.g. precipitation), and so on. Initial analysis indicated that the relationship between dbh and mortality was U-shaped, corresponding to high mortality in small trees, low mortality for medium sized trees (typically 25–40 cm) and increasing mortality in larger trees. To describe this relationship we tried several different model equations and found the best fit to the data using the following functional form
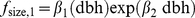
(3)where 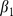 and 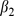 are parameters. In keeping with the qualitative pattern visible in the initial assessment of the size-dependency of mortality, Eqn (3) allows the initial decrease in mortality vs. size for small trees to be steeper than the increase in mortality for size for larger trees whilst giving high flexibility to the shape of the response. For each environmental variable *V* (ie climate and soil measures) we considered two alternative functional forms:
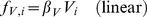
(4)

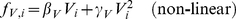
(5)where *V_i_* is the value of environmental variable *V* associated with tree *i*, and 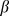 and 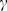 are parameters. We used the same functional forms to include the effects of plot basal area *B* (m^2^ ha^−1^):
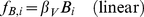
(6)

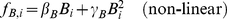
(7)where *B_i_* is the plot basal area *B* associated with tree *i*, and 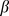 and 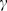 are parameters. Although we chose to use a quadratic functional form, we did not constrain the shape further so that, within a species' range, it could predict shallow or steep monotonic curves, as well as U-shaped (or hump-shaped) responses. Since we had no strong evidence for a particular across-species response for any of the environmental variables we felt that a quadratic functional form would be sufficiently complex to capture essential patterns without being too complex. Together, Eqns (1)–(7) allow for a very large possible number of models, with a wide variety of numbers of parameters, depending on which predictor variables are included, and depending on whether each variable is included using a linear or non-linear (quadratic) functional form. We allowed each parameter in any given model to be either species-specific (e.g. in Eqn (4) this would give us 21 separate 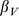 parameter values, one for each species, each of which is unaffected by data from other species) or global, that is, shared among species (e.g. in Eqn (4) there would be a single 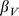 value for all trees regardless of their species). To avoid having to fit all possible models, we used a selection procedure that compared models with major differences in their predictor variables (see *model selection*, below).

### Parameter estimation

We used Bayesian methods based on Metropolis-Hastings Markov Chain Monte Carlo sampling [41] to estimate values and confidence intervals for each of the parameters in each model. These methods were chosen because they allow for simple, efficient estimation of parameters, including confidence intervals. However, we did not use informative priors, so the outcome of the analysis can be expected to be similar to the outcome of a Maximum Likelihood analysis using the same data and models. The first step of the analysis was to define, for a given candidate model *M*, the log-likelihood of the inventory data (referred to here as *X*), given a particular set of parameters (referred to here as 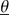) values for model *M*:
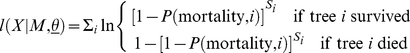
(8)Eqn (8) represents a sum, over all trees *i*, of the logarithm of the probability of the observation for *i* (survived or died), given the model structure *M* and parameter set 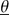, where *S_i_* is the survey interval (years) for tree *i*.

We used non-informative uniform priors on all parameters so the MCMC algorithm (see below) needed to refer to the log-likelihood only. However, for numerical reasons we imposed upper and lower limits on the allowable values of all parameters, i.e., a prior probability of 0 on parameter values outside of the allowable range. We set the allowable range much wider than the plausible values, and also checked the posterior distributions to make sure the tails of the posterior distributions were a long way from the edge of the allowable range.

The next step was to estimate values for the parameter set 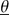 in model *M*, given the definition of the log-likelihood (Eqn (8)). We did this using an adaptive Metropolis MCMC algorithm [42], [41], which returns random samples from the posterior distribution of 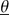. At each iteration, a particular parameter *p_k_* is chosen and altered by adding a random value from a normal distribution 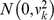 where *v_k_* is specified for each parameter. The likelihood of the data given the new parameter is calculated and the parameter change is ‘accepted’ based on the ratio of the new likelihood and the previous likelihood:
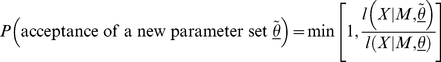
The variance *v_k_* for each parameter was tuned during a ‘burn-in’ period to achieve an optimal parameter acceptance rate of 25% [41] so the samples returned from the MCMC can be said to have efficiently sampled the posterior of each parameter.

We implemented the MCMC algorithm by initializing each parameter value at a random point close to the middle of the allowable range, allowing a suitable burn-in period (between 25,000 and 1,000,000 iterations) for the algorithm to reach quasi-equilibrium, then recording every 100^th^ sample of 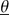 (to avoid auto-correlation) from a post burn-in period of between 50,000 and 250,000 iterations (the number required depended on the speed of model convergence and the number of parameters). This provided us with a set of between 500 and 2,500 samples of 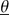 for each model *M* that we parameterized. From these samples, we calculated the mean, and 95% confidence interval, of each parameter *p* within 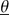. For the best-fit model we re-ran the model four times with differing starting parameter values and found the results were unchanged.

### Model selection

As metrics to compare alterative models, we calculated, for each model *M* that we parameterised, the Akaike Information Criterion (AIC; [43]) and the Bayesian Information Criterion (BIC; [44]). Both criteria reward models for providing a better fit to the data, but penalise models according to the number of free parameters that they contain, thus allowing for model selection from sets of models that differ in model complexity. However, the AIC penalises complexity less strongly than the BIC, so it is useful to compare the two criteria. Simple likelihood-ratio based comparisons would not have been appropriate since the models were, in general, non-nested [45]. Both criteria require an estimate of the maximum likelihood, for which we used the maximum value of the log-likelihood encountered by the MCMC algorithm in the post burn-in period.

Given the high number of possible mortality predictors, the options of functional forms presented by Eqns (2)–(7) and the choice of species-specific or global for any parameter, there was a very large set of possible models *M*. We wished to select an appropriate best model from this set, but without having to examine every possible combination of possible predictors. To do this we used the procedure outlined in the next three paragraphs.

First, we established which of the possible predictor variables was the best single predictor of mortality by parameterising all possible mortality models featuring one predictor variable (referred to here as 1-d models). This set of models was still relatively large (28 different models), since the predictor variable in question could included using a linear or non-linear function, and with species-specific or global parameters (see Eqns (4)–(6)). We also tested some of the closely correlated alternative climate predictors in this way, but none gave a better fit than the set we had already chosen. Comparing the AIC and BIC values associated with each model allowed us to determine whether, considered in isolation, each predictor variable was best described using species-specific vs. global parameters, and a linear vs. non-linear functional form (see Eqns (4)–(6)). This analysis suggested that all predictor variables were best described using non-linear, species-specific functional forms. Therefore we decided to retain, within the larger set of all possible models, only those models that included non-linear functional forms. Further, comparing the maximum likelihood of the different 1-d models allowed us to rank the predictor variables in descending order of importance (meaning importance considered in isolation). The rank was: size>>radiation>yearly precipitation>mean annual temperature>plot basal area>maximum wind speed>soil type. Since size (dbh) was by far the best single predictor of mortality, we decided at this point to discard, form the large set of all possible models, any models not including dbh as a predictor variable.

Second, we sought, within the remaining set of models, the best set of environmental variables to include in the model. Since radiation was the best single environmental predictor, we tested each additional environmental predictor to find the best two-predictor combination, using species-specific responses, giving a model of the form:
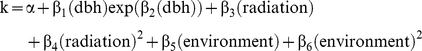
We found that adding in yearly precipitation gave the best fit. We repeated this procedure to find the best three and four parameter models, and finally checked that including all five predictors (radiation, yearly precipitation, mean annual temperature, maximum wind speed and soil type) gave a better fit than the other models, using AIC and BIC.

These steps gave us for types of predictor variable: the constant α (Eqn (2)), dbh (Eqn (3)), the set of five non-linear environmental effects (Eqn (5)) and the non-linear competition effect (plot basal area: Eqn (7)). To determine the final model form we generated a set of models which allows us to test whether each type of predictor should be species-specific or shared, and whether the extra model complexity added by including environmental and competition effects in the simple size model was justified by the improvement in fit. We tested models using every combination of species-specific or shared effects for each type of predictor, as well as every combination with or without environment and competition effects (36 models in total). The full list of different models tested are shown in Table S1 (see Supporting Information), along with AIC and BIC scores. The score of the best model was a very large improvement on the next best, although it is worth noting that models without environmental effects performed significantly worse than those without plot basal area as a predictor.

### Parameter significance

The majority (74%) of parameters' 95% posterior distributions did not include 0, indicating statistically significant effects for these parameters. None of the posterior distributions for the constant or size parameters (α_j_, β_1j_ and β_2j_ in Eqn (4)) included 0, while the least significant deviations from zero were seen for soil type and maximum wind speed parameters, and for the species *Liquidambar styraciflua*, *Thuja occidentalis* and *Nyssa slyvatica*. In principle many additional parameterizations could be used to eliminate some effects for some species (i.e. remove terms associated with species-parameters with posteriors including zero), but we considered that this extra computational effort could not be justified in terms of increased scientific understanding.

### Interpretation of the results

In order to compare the different mortality rates predicted for each species we calculated a single ‘baseline’ mortality of each species as the predicted mortality of a tree of standard size growing in a standard environment (we used both the mean environment, taken over the study region, for all variables together with a ‘mesic’ soil texture; and the species' own median environment). We chose to use 20cm as the standard stem diameter because it is approximately the size of a canopy tree [3].

In order to visualise geographical patterns in observed mortality rates, we calculated a mortality rate for each plot (“plot-averaged mortality”) by fitting a single-parameter logistic model to the data, and used the coordinates of each to create a regional mortality map. We visualised geographical patterns in predicted mortality rates by creating simulated datasets which were identical to the original dataset except that whether each tree died or not was determined using the model's posterior parameter values. We then used the simulated data to calculate a model-predicted mortality rate for each plot. For each tree *i* we calculated its annual mortality rate based on the model equation generated from the randomly chosen parameter set. From this we determined the probability it died, *P_i_*, over the whole survey period, and then assigned it as dead with probability *P_i_* and alive with probability 1- *P_i_* within the simulated dataset. We generated 100 simulated datasets in this way, using different parameter sets randomly drawn from the joint posterior of the parameters, and combined their results using likelihood profile methods to predict a single model-predicted mortality rate for each plot. Using multiple randomly chosen samples from the joint posterior instead of simply the mean value of each parameter accounts for any co-variance in the parameters and the effect this would have on the predictions. By comparing the predicted and observed maps we were able to examine how well our predictions fitted the observed mortality and in which regions there were mismatches.

We also wanted to create maps showing how mortality varies regionally in response to variation in species identity, stand structure (stem size and plot basal area) and environmental conditions, whilst controlling for variation in the other factors. We devised an approach to do this, based on creating simulated datasets in various different ways which selectively removed *variation* in the predictors which were not of interest. For example, to analyse the mortality patterns arising from variation in stand structure, we generated new 100 simulated datasets in which the tree alive/dead column was predicted from our model by using size and plot basal area, but assuming all trees were *Acer rubrum* (the most common and wide-ranging species in the dataset) and every tree experienced the same environmental conditions (the region's average). Similarly, to analyse mortality patterns arising from species composition, we retained species information but assumed all trees had the same size (20cm dbh), basal area (the average density) and environmental conditions (the region's average) when creating the dead/alive column of the simulated datasets. Finally, to analyse mortality patterns arising from variation in environmental conditions we retained environmental information but assumed all trees were *A. rubrum*, and had the same size (20cm dbh) and were in plots of average density.

Our maps are an imperfect way to partition spation variation but this method allows us to analyse variation in mortality due to each factor by selectively controlling for variation in the others. Had we chosen a different size of tree or a different set of environmental conditions, we would have seen the same spatial variation in mortality rates but the overall level of mortality would have been different. Since different species responded in different ways to changes in environment and stand structure, we also calculated variation in mortality due to these factors but using *Pinus taeda* (the most common gymnosperm species) instead of *A. rubrum*. However, this species has a much smaller range than *A. rubrum* so we only considered variation in mortality in the region in which the species is found.

We were also interested in seeing how mortality varied along the range of each predictor, both for all species together (“forest-averaged mortality”) and for each individual species (“species-averaged mortality”). We generated estimates of how observed mortality varied along the range of each predictor by binning the raw data according to the predictor of interest into equal sized bins (i.e. each containing the same number of stems) and found the best single annual mortality rate for the whole bin in the same way as before, using a single parameter logistic model. We did this both for the raw data (for just the 21 species for which we parameterised the model) and for all the data (including the rare species). In order to compare this to the model predictions for all species together (the forest-averaged mortality) we created 100 sets of simulated data as before (i.e. data of the same form as the original dataset but with alive/dead status based on our model predictions), ordered and binned these according to the variable of interest and calculated a single mortality rate for each bin, and a 95% confidence interval on this rate. Thus the forest-averaged mortality accounted for simultaneous changes in species composition and size structure across whichever gradient was being considered, and could be compared to the observed data.

Finally, for each species we were interested in how mortality varied with changes in the variable of interest alone, but since the predictors (size, environment and stand basal area) all co-varied along each gradient we calculated the median conditions in which each of the species was found. For each model predictor we created 100 simulated datasets using parameter values randomly chosen from the joint parameter posterior distributions. In these datasets, each tree was given the median condition of its species (apart from the predictor of interest) and was assigned as dead or alive based on its predicted annual mortality rate. For example, in order to examine the sole effect of temperature change on mortality we re-assigned each tree the median size, precipitation, radiation, maximum wind speed, soil type and stand basal area in which its species was found in the original dataset, and kept only the temperature information for each individual tree and then created the 100 simulated datasets as before by selecting 100 parameter sets at random from the joint posterior. This gave us a spread of mortality vs. temperature functions for each species, where the spread represents parameter uncertainty (variation in parameters causing variation in probability of mortality) and sampling (random variation in whether lived or died given the probability of mortality). This allowed us to consider only the effect of temperature on that species mortality, whilst modelling the species in a reasonable environment.

## Results

### Model selection

**Figure 1:**
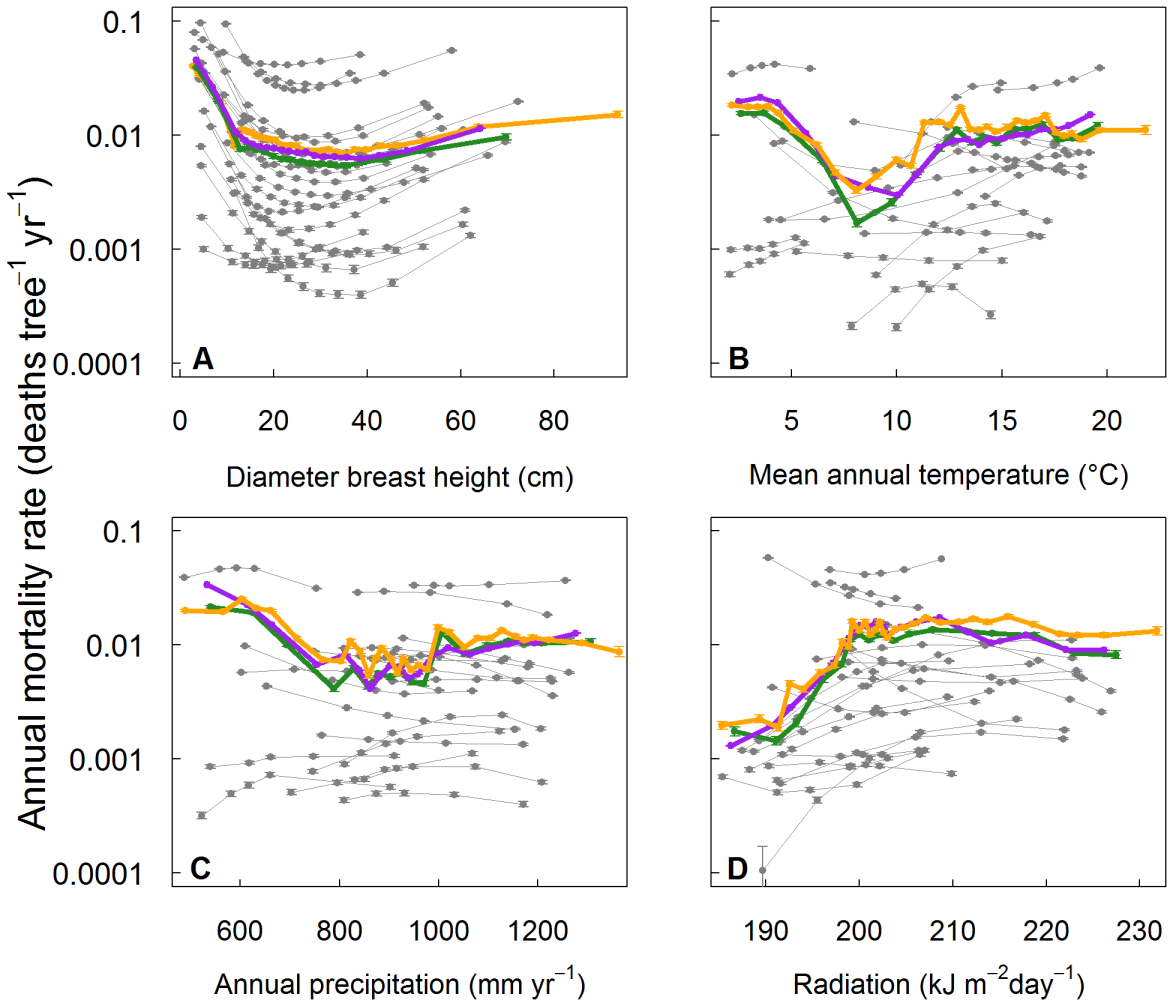
Observed and predicted mortality against stem size and environmental gradients. Observed and predicted forest-averaged and species-averaged annual mortality rates (deaths tree^−1^ yr^−1^, log scale) plotted against (**A**) diameter at breast height (cm), (**B**) mean annual temperature (°C), (**C**) total annual precipitation (mm/year), and (**D**) solar radiation (kJ m^−2^ day^−1^). Each panel shows the observed trends in mortality calculated using data from all species (orange) and from the 21 most common species (green), and the predicted curves for 21 common species (grey) and the combined curve from these species (purple). Individual species mortality rates are shown vs. changes in the predictor variable of interest alone, i.e. with all other predictor variables held at the median for that species (see Supporting Information). Error bars on the predictions (grey and purple) are 95% confidence intervals calculated from an error propagation procedure that accounted for parameter uncertainty. Error bars on the observations for the whole forest including rare species (orange) and 21 species combined (green) are 95% confidence intervals for mortality rates in the data (see Supporting Information).

Using AIC and BIC, we found that the 7 best performing models all included species-specific environment effects, even when other predictors were not species-specific, or when plot basal area was not included. Plot basal area was only found to be a worthwhile predictor if its effects were species specific but did not benefit the model if the effect was shared among species. Models with non species-specific constant or size effects performed well, but the model with all predictors included as species-specific performed significantly better than all the others, according to both AIC and BIC. Therefore in our final model the function k (Eqn (1)) took the form:
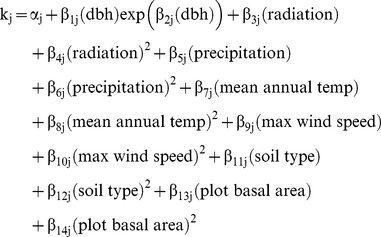
(9)where j is the species and α_j_ and the β_j_s were the parameters estimated (so a different function k_j_ was estimated for each species). The MLEs, Bayesian means and confidence intervals for the parameters for each species of the best fit model (Eqn (9)) are given in Table S2 (see Supporting Information). The predicted trends in mortality were close to the observed patterns across all predictor variables included in the model (Fig. 1, S1, S2, S3 in Supporting Information) suggesting that the structure of the model was appropriate for capturing mortality patterns within these data.

### Species-mortality relationships

**Figure 2:**
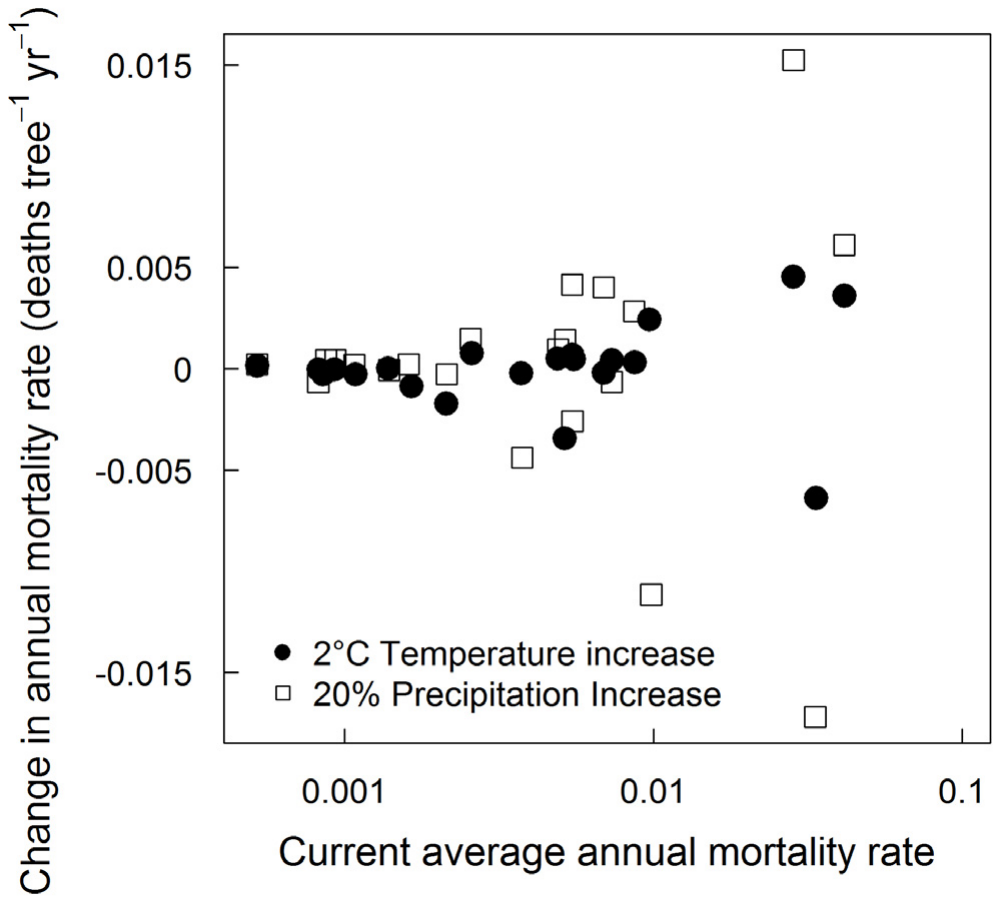
Predicted changes in species' mortality rates with increases in temperature and precipitation. Predicted changes in species' average annual mortality rate (calculated at each species' median size and environment) when subjected to a hypothetical 2°C temperature increase (•) and a 20% increase in annual precipitation (□), shown plotted against the current average mortality rate without this change.

Species showed very different baseline mortality rates, even when other effects were factored out (Table 1), and as a consequence plot-level mortality is highly sensitive to species composition. To illustrate this point we compared species mortality rates calculated at the median environment of each individual species. These mortality rates differed widely: the highest, for *Populus tremuloides*, was 80 times larger for than for the lowest, *Quercus prinus* (Table 1). In addition to the differences in baseline mortality, species showed contrasting responses to environmental variation. For example, the model predicts substantial species differences in the direction and magnitude of responses to hypothetical increases in temperature and precipitation (Fig. 2).

**Table 1:** Species' predicted annual mortality rates.

	Mortality in forest mean environment		Mortality in each species' median environment	
Species	Annual mortality rate	95% CI	Annual mortality rate	95% CI
*Acer rubrum*	0.0035	(0.0034, 0.0038)	0.0022	(0.0020,0.0023)
*Acer saccharum*	0.0108	(0.0104, 0.0112)	0.0052	(0.0049,0.0054)
*Betula papyrifera*	0.0012	(0.0010, 0.0014)	0.0009	(0.0008,0.0011)
*Carya spp*	0.0011	(0.0009, 0.0012)	0.0026	(0.0023,0.0028)
*Fagus grandifolia*	0.0020	(0.0018, 0.0022)	0.0017	(0.0014,0.0019)
*Fraxinus americana*	0.0016	(0.0014, 0.0018)	0.0008	(0.0007,0.0010)
*Liquidambar styraciflua*	0.0040	(0.0037, 0.0042)	0.0048	(0.0045,0.0052)
*Liriodendron tulipifera*	0.0005	(0.0004, 0.0006)	0.0009	(0.0008,0.0010)
*Nyssa sylvatica*	0.0180	(0.0170, 0.0187)	0.0323	(0.0311,0.0336)
*N. sylvatica (biflora)*	0.0044	(0.0040, 0.0049)	0.0098	(0.0090,0.0104)
*Populus tremuloides*	0.0017	(0.0015, 0.0018)	0.0407	(0.0399,0.0414)
*Quercus alba*	0.0016	(0.0015, 0.0017)	0.0013	(0.0012,0.0014)
*Quercus nigra*	0.0094	(0.0088, 0.0102)	0.0068	(0.0063,0.0074)
*Quercus prinus*	0.0004	(0.0003, 0.0005)	0.0005	(0.0004,0.0006)
*Quercus rubrum*	0.0062	(0.0059, 0.0064)	0.0035	(0.0033,0.0038)
*Quercus stellata*	0.0392	(0.0384, 0.0399)	0.0083	(0.0078,0.0088)
*Quercus velutina*	0.0114	(0.0110, 0.0119)	0.0072	(0.0068,0.0075)
*Pinus echinata*	0.0157	(0.0151, 0.0163)	0.0054	(0.0051,0.0057)
*Pinus taeda*	0.0020	(0.0018, 0.0022)	0.0054	(0.0052,0.0057)
*Pinus virginiana*	0.0047	(0.0042, 0.0052)	0.0282	(0.0271,0.0299)
*Thuja occidentalis*	0.0114	(0.0110, 0.0119)	0.0011	(0.0010,0.0013)

Predicted baseline annual mortality rate (deaths tree^−1^ year^−1^) calculated for each species both in the mean environment of the dataset for 20cm dbh trees, and at each species' median environmental conditions (that is the conditions at which the highest number of individuals within the dataset were found), using the best fit model. 95% confidence interval for the mortality rates are also given.

### Size-mortality relationship

The relationship between size (dbh) and mortality was U-shaped for all species (Fig. 1A: p<<0.001 for all species). The highest mortality rates were found for the smallest trees and the lowest rates for trees of 18–37 cm dbh. The rate at which mortality decreased with size in saplings and increased with size in larger trees varied considerably among species, from relatively flat (e.g. *Thuja occidentalis*) to dramatic (e.g. *Acer saccharum*). However, species with higher minimum mortality consistently showed both higher sapling mortality and higher mortality at larger stem sizes (Spearman's Rank Correlation p<0.05 for both trends). Forest-averaged mortality was U-shaped mortality vs. size (i.e. when all data were grouped together, the model applied to each individual and the total average mortality calculated). However, the upturn in forest-averaged mortality in large trees was less pronounced as larger size classes became increasingly dominated by species with low mortality rates.

### Environment-mortality relationships

Of the several environmental factors included in the model, temperature and precipitation are particularly important in this region because they are likely to change substantially, and perhaps rapidly, under anthropogenic climate change [46]. Forest-averaged mortality was U-shaped against annual mean temperature. The minimum mortality, which occurred at a temperature of around 8–10°C, was 6–9 times lower than the rate at low or high temperatures (mean annual temperature <5 or >15°C) (Fig. 1B). This observed pattern was robust to whether all species, or only the 21 common species, were considered (Fig. 1B, green and orange lines respectively), and was reproduced by our model (Fig. 1B, compare green and purple lines). This occurred despite the fact that the observed forest-averaged mortality pattern across the temperature gradient was not always reflected in the species-averaged responses of the particular species present at those temperatures. In the range 10–15°C, both the forest-averaged mortality and the species-averaged mortality for the majority of the species increased with temperature (Fig. 1B, grey lines). However, forest-averaged mortality decreased with increasing temperature below 10°C and increasing mortality with increasing temperature above 15°C. In contrast, species-averaged mortality for the majority of species found in these temperature ranges showed the opposite trend. Analogous mismatches in the response of particular species vs. the forest average were also found for precipitation (Fig. 1C) and radiation (Fig. 1D).

Forest-averaged mortality rates decreased with increasing precipitation up to a threshold of around 800 mm yr^−1^ and showed no clear trend thereafter (Fig. 1C), but individual species showed both increasing and decreasing mortality in the driest part of the range. At higher precipitation levels the forest-averaged mortality pattern was less clear, with some species showing increasing species-averaged mortality with higher precipitation (producing an overall U-shaped response to precipitation), and some a flat response. The opposite effect was found in the relationship between mortality and radiation, with a strong trend for increasing forest-averaged mortality up to a threshold point of about 200 kJ m^−2^ day^−1^ (Fig. 1D), after which the response was much flatter. For most species we found that species-averaged mortality vs. basal area was hump-shaped (p<0.05 for 16 of 21 species), with 50% of species showing maximum mortality in stands of 10–37 m^2^ ha^−1^ (Fig. S3 in Supporting Information). The inclusion of less common species raised the observed forest-averaged mortality rate, but otherwise left the patterns unchanged (Fig. 1 and see Supporting Information Fig. S1, S2, S3).

### Geographical variation in mortality

**Figure 3:**
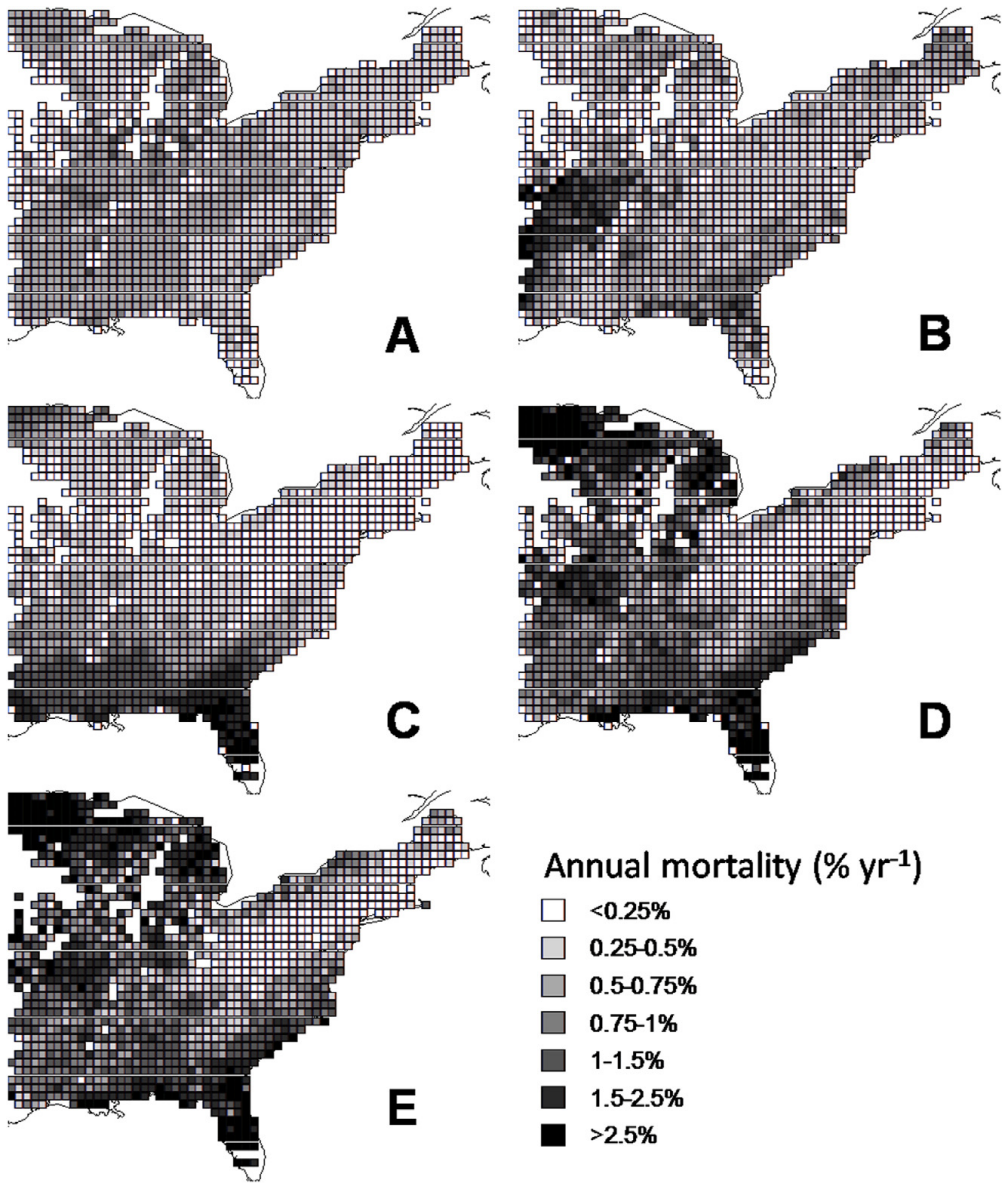
Regional variation in mortality due to variation in each component of the model. Maps of estimated annual forest-level mortality across the Eastern United States illustrating the contributions of each of the components of the model (**A–C**), the full model results (**D**), and the observed mortality for the 21 common species (**E**). (**A**) Variation in forest structure alone (stem size and plot basal area), illustrated by removing environmental effects and modelling just the most common species (*A. rubrum*). (**B**) The effect of variation in forest species composition alone, illustrated by removing environmental variation, and stand structure variation (i.e. modelling a 20 cm dbh tree). (**C**) The effect of variation in environment alone, illustrated by modelling *A. rubrum* without stand structure variation (i.e. modelling a 20 cm dbh tree) across the region. Full model results (**D**) are strongly affected by species-environment interaction, and closely match the observed geographical pattern of average mortality (**E**).

The model reproduces most of the geographical patterns in plot-averaged mortality observed in the FIA dataset (compare Fig. 3D and Fig. 3E) with a high correlation seen between observed and predicted mortality in plots with more than 10 stems (Figure S4: r^2^ = 0.89). Since the model reproduced geographical variation well, we were able to decompose the variation into the separate effects of stand structure (stem-size distributions and plot basal area), environment and species (Fig. 3A–C). According to this decomposition, variation in species composition and environmental conditions were much more important than variation in stand structure in determining geographical patterns in plot-averaged variation in mortality. High observed plot-averaged mortality in the southeast is reproduced by considering only the environmental conditions of the region, but not when only stand structure or species composition are considered (Fig. 3C). In particular, several species common in the southeast (e.g. *Nyssa sylvatica*, *A. rubrum* and *Quercus nigra*) showed strongly increasing species-averaged mortality with the higher average temperatures, but this effect doesn't appear when only species composition is considered since temperatures in the region are much higher than these species' median environments. High plot-averaged mortality in the west is driven primarily by species composition (Fig. 3B); whilst variation in stand structure has relatively little impact on plot-averaged across the region (Fig. 3A). We checked whether our conclusions were dependent on our choice of species (i.e. *A .rubrum*) by creating the equivalent maps using the most common gymnosperm species, *P. taeda* (Fig. S5). We again found that variation in environmental conditions resulted in higher variation in mortality than variation in stand structure (Fig. S5).

However, not all variation predicted by the model was explained by a simple sum of the three components, indicating strong interactions between them. For example, both stand structure and species composition (Fig. 3A, 3B) predict higher plot-averaged mortality in the northeast than is predicted by the model or is observed (Fig 3D, 3E), indicating an interaction with environmental conditions (Fig 3C), which predict lower plot-averaged mortality in the area. The largest differences between model predictions and observations of plot-averaged mortality were all in plots with less than 100 stems. Differences were mostly due to underestimated plot-averaged mortality by the model, particularly in the furthest northwest and southeast of the region where many plots were dominated by species too rare across the whole region to be included in our analysis (see Supporting Information Fig. S6).

## Discussion

### Size and stand structure

We found that size (dbh) was the single variable with the greatest effect on mortality rate at the level of the individual tree, with trees of intermediate size exhibiting mortality rates much lower than smaller, or larger, trees. This U-shaped relationship between size and mortality appears to be a common feature of forests, whether from sub-boreal [47], temperate [48]–[50] or tropical [51] regions. It seems likely that this feature results from two opposing effects: (i) mortality is often high when trees are small because they are competitively inhibited by taller neighbours, but with higher light levels show an increase in growth rate and reduction in mortality [2]; and (ii) a general increase in mortality in larger individuals due to senescence and/or increased exposure to strong wind and other disturbance agents [52], [53], [50]. This explanation is supported by the fact that species exhibit their minimum mortality rates at around the size they enter the canopy (around 20 cm dbh, corresponding to a height of around 20 m for a typical Eastern US deciduous tree; [3]): once in the canopy, individuals are less affected by competition for light with neighbours. U-shaped mortality has potentially major implications for understanding forest structure and the forest carbon cycle, because larger trees contain a disproportionate fraction of above-ground woody biomass, such that any increase in their mortality has a large effect on carbon storage [7].

However, despite size being the most important single predictor of mortality at the tree scale, variation in stand size structure had almost no effect on geographical variation in plot-averaged mortality (Fig. 3A). This may be simply because stand structure does not vary systematically across the region, otherwise, geographical variation in size distributions would result in geographical variation in plot-averaged mortality. However, the precise way in which the dynamics of size distributions might interact with climate change and/or changes in tree harvesting to induce future changes in plot-averaged mortality remains largely unexplored.

### Within vs across species variation in mortality along climatic gradients

The mismatches we found between species-averaged and forest-averaged mortality -environment correlations imply that, under climate change, forest-averaged mortality will change in ways that cannot be anticipated by examining the current relationship between observed mortality and climate. Given that mortality is highly dependent on species identity, size and environmental factors, it is important to include all these factors in predictive models of climate-change effects. For example, consider the response of carbon stocks in the coldest regions of the Eastern US to a scenario of increased temperature. Forest-averaged mortality is currently greatest in the coldest locations, suggesting that warming should decrease mortality rates, and increase carbon stocks (Fig. 1B). In contrast, the fact that the species that currently dominate cold regions had higher species-averaged mortality rates in warmer areas implies that the warming might increase mortality in cooler regions dominated by these species. Although warming-induced mortality increases have been observed in other temperate forests [8], [54], even this extrapolation must be viewed with caution, because it ignores any simultaneous changes in species composition.

At the forest-averaged level, wind speed did not have an effect on mortality yet several species-averaged mortality rates showed a strong correlation with it (Fig. S1). The effect may be confounded with other variables, for example trees may experience higher mortality with higher wind speed in low density areas where there is little protection from neighbouring trees [55]. At the forest-averaged level, we found that mortality increased with increasing radiation (Fig. 1D). A similar pattern of increasing mortality in higher light conditions has been found for oak seedlings in the Mediterranean [56] and linked to higher desiccation risk. Although several species followed this pattern, many others showed the opposite trend of decreasing mortality with increasing radiation, in agreement with many other studies linking light to survival (e.g. [57]–[59]).

### Species-mortality responses to changing climate

Our results suggest that species show contrasting responses to changing environmental conditions, and these mortality responses were strongly non-linear which suggests that individuals within a species may respond at different rates to a change in conditions, depending on where they sit within the species range. Changes in mortality have been correlated with changing temperature and precipitation levels in the USA in other studies [60], [54], and since many parts of the Eastern USA are predicted to experience increases in temperature and precipitation under climate change [46], we examined changes in mortality under scenarios of blanket increases in temperature and precipitation only (Fig. 2). We found that the largest changes were seen in the species with the highest mortality rates, implying that under these climate change scenarios the largest changes in carbon dynamics might be seen in highly-disturbed landscapes where fast-growing species dominate. Such changes in mortality could have repercussions for forest structure and species composition, but any consequences would need to be understood in the context of compounding effects of species-specific changes in growth and recruitment rates [61], [11], and frequency of disturbance events, such as pest and pathogen outbreaks, which may change with climate change [62]. However, since observed wood anatomy and demographic rates within a species may have adapted to local climatic conditions [63], the future response of mortality to rapid climate change may follow different patterns to the correlations between chronic climatology and mortality documented here.

### Limitations

Although this work presents strong evidence for marked variation in mortality with a variety of different factors, we recognise several shortcomings in terms of a lack of inclusion of external disturbance factors, forest management and history, which are all likely to affect mortality. It is also important to note a significant limitation of the study, namely that the data used cover a single survey period only (1980s–1990s), so particular quantitative results are dependent on conditions in this period and must be treated with caution. This raises the possibility that some of the patterns reported here reflect particular episodic events that may not be representative of mortality patterns averaged over the longer term. However, our four main conclusions (that mortality is U-shaped against dbh, hump-shaped against plot basal area, and species exhibit both different underlying mortality rates and different responses to changes in environmental conditions) presented in the main paper are robust unless: (a) over longer periods temporal variation completely or nearly removes all effects of species, size or environment on mortality, (b) the apparent effects of the different predictor variables on mortality uncovered here were caused *entirely* by temporally varying factors not considered by this study, for example pests and pathogens [17], forest management practices or extreme weather events [64]. Fortunately, national forest inventories are beginning to provide re-surveyed data covering more than one time interval (e.g. [40]). In principle, this kind of data can be used to estimate the magnitude of inter-decadal variation in tree mortality directly. These limitations are important and call for caution in interpreting the results given here, and/or in utilising our models of mortality (Table S2 in Supporting Information). More importantly, these limitations, together whether the marked correlations between climate and mortality documented here, call for further research into tree mortality and its potential contribution to the response of the terrestrial carbon cycle to climate change.

### Conclusion

We found large and statistically significant differences in mortality among species not only in baseline mortality rates (Table 1), but also in their responses to environmental variation (Fig. 1, S1, S2, S3 in Supporting Information), along with marked effects of individual size, and plot basal area. Importantly, both species composition and stand structure are likely to continue to undergo directional changes over decadal timescales, independent of any effects of climate change. Therefore, projections of future forest carbon dynamics will be in error unless they incorporate the effects of projected changes in species composition and stand structure. The good news is that recent decades have seen the appearance of a variety of simulation models that can make accurate predictions of forest dynamics, whether within the context of forest community ecology (e.g. [3]) or silviculture (e.g. [65]), as well as Dynamic Global Vegetation Models which in principle can predict forest responses to changing CO2 concentrations (e.g. [66]). These models, together with the large forest inventory databases that are rapidly becoming available for many of the world's forests, suggest that believable predictions of future forest dynamics and the forest carbon balance are within reach.

## Supporting Information

Table S1 Table comparing model fits using AIC and BIC 36 models were run within which the four types of model predictor in Eqn (4) (constant, size, environment, basal area) were left out or included with forest-level (FL) or species specific (SS) effects. Total number of parameters, AIC and BIC scores and rankings are reported. Models without size and species effects were rejected very strongly, and the additional inclusion of environmental and competition variables increased model fit significantly. The best-fit model, number 26, showed a very significant improvement on the next best using both AIC and BIC.
(0.07 MB DOC)

Table S2 Table of maximum likelihood estimators (MLEs), Bayesian means and 2.5% and 97.5% confidence levels calculated from the posterior distributions for each of the 15 parameters of Eqn (9) for each of the 21 common species parameterised by the adaptive MCMC algorithm. The burn-in for the algorithm was 750,000 iterations and the sampling was 250,000 iterations.
(0.18 MB DOC)

Figure S1 Observed and predicted mortality rates against maximum wind speed. Log annual mortality rates observed for the whole forest including rare species (orange) and the 21 common species (green), and the model predictions for the 21 species combined (purple) and each species individually (grey), plotted against maximum wind speed (m/sec). Species' error bars (grey) show parameter uncertainty, forest error bars (purple, orange and green) show the 95% confidence interval for the mortality rates predicted from the model-created and real datasets.
(8.20 MB TIF)

Figure S2 Observed and predicted mortality rates against soil type. Log annual mortality rates plotted against soil type for the predicted forest-level mortality rate for all 21 species parameterised by the model (purple), the real forest-level mortality rates for the 21 species (green) and the whole forest including rare species (orange). Error bars (purple, orange and green) show the 95% confidence interval for the mortality rates predicted from the model-created and real datasets.
(8.20 MB TIF)

Figure S3 Observed and predicted mortality rates against plot basal area. Log annual mortality rates observed for the whole forest including rare species (orange) and the 21 common species (green), and the model predictions for the 21 species combined (purple) and each species individually (grey), plotted against plot basal area (m^2^/hectare). Species' error bars (grey) show parameter uncertainty, forest error bars (purple, orange and green) show the 95% confidence interval for the mortality rates predicted from the model-created and real datasets.
(8.20 MB TIF)

Figure S4 Observed versus predicted plot-averaged mortality rates. Observed versus predicted plot-averaged annual mortality rate for all plots with at least 10 stems, showing the high correlation (r^2^ = 0.9).
(8.20 MB TIF)

Figure S5 Patterns of mortality due to regional variation in stand strucuture and environmental alone. Maps of estimated annual forest-level mortality across the Eastern United States illustrating the contributions of variation in stand structure (stem size and plot basal area) and environment, modelled across the range of *Pinus taeda* to control for the effects of species composition. (A) Variation in forest structure alone (stem size and plot basal area), illustrated by removing environmental effects and modelling just the most common species (*P. taeda*). (B) The effect of variation in environment alone, illustrated by modelling *P. taeda* without stand structure variation (i.e. modelling a 20 cm dbh tree) across the region.
(8.88 MB TIF)

Figure S6 Regional patterns of differences between observed at predicted mortality rates. Map of absolute difference between predicted and observed forest level mortality across the Eastern United States.
(7.66 MB TIF)
