# Supplementary material for: Influences of Forest Structure, Climate and Species Composition on Tree Mortality across the Eastern US
Source: PLoS One. 2010 Oct 13;5(10):e13212. doi: 10.1371/journal.pone.0013212 (PMC2954149; doi:10.1371/journal.pone.0013212)
Supplement: Table S1 — Table comparing model fits using AIC and BIC 36 models were run within which the four types of model predictor in Eqn (4) (constant, size, environment, basal area) were left out or included with forest-level (FL) or species specific (SS) effects. Total number of parameters, AIC and BIC scores and rankings are reported. Models without size and species effects were rejected very strongly, and the additional inclusion of environmental and competition variables increased model fit significantly. The best-fit model, number 26, showed a very significant improvement on the next best using both AIC and BIC. (0.07 MB DOC) [file pone.0013212.s001.doc]

**Table S1. Comparison of 36 models tested using AIC and BIC.**

| **Model**  **Number** | **Constant** | **Size** | **Environment** | **Basal Area** | **# parameters** | **AIC score** | **AIC rank** | **BIC score** | **BIC rank** |
| --- | --- | --- | --- | --- | --- | --- | --- | --- | --- |
| **1** | FL | FL | - | - | 3 | 285046.8882 | 37 | -142540.0664 | 37 |
| **2** | FL | SS | - | - | 47 | 250075.4929 | 26 | -125298.1627 | 23 |
| **3** | SS | FL | - | - | 25 | 250214.3687 | 27 | -125245.7036 | 21 |
| **4** | SS | SS | - | - | 69 | 249993.7152 | 24 | -125379.1708 | 24 |
| **5** | FL | FL | FL | - | 13 | 271511.4679 | 35 | -135827.764 | 34 |
| **6** | FL | SS | FL | - | 57 | 243591.2996 | 12 | -122111.4737 | 8 |
| **7** | SS | FL | FL | - | 35 | 248196.538 | 18 | -124292.196 | 17 |
| **8** | SS | SS | FL | - | 79 | 243336.1548 | 11 | -122105.7983 | 7 |
| **9** | FL | FL | SS | - | 233 | 242284.941 | 9 | -122433.4701 | 9 |
| **10** | FL | SS | SS | - | 277 | 238863.244 | 4 | -120966.4155 | 4 |
| **11** | SS | FL | SS | - | 255 | 241014.0694 | 6 | -121919.9312 | 6 |
| **12** | SS | SS | SS | - | 299 | 237455.7338 | 3 | -120384.5574 | 2 |
| **13** | FL | FL | - | FL | 5 | 282448.4993 | 36 | -141251.9535 | 36 |
| **14** | FL | SS | - | FL | 49 | 250312.1483 | 28 | -125427.5719 | 25 |
| **15** | SS | FL | - | FL | 27 | 250028.5919 | 25 | -125163.8967 | 20 |
| **16** | SS | SS | - | FL | 71 | 249753.8625 | 22 | -125270.326 | 22 |
| **17** | FL | FL | - | SS | 49 | 253033.1349 | 30 | -126788.0652 | 30 |
| **18** | FL | SS | - | SS | 93 | 248935.0076 | 20 | -124982.7955 | 19 |
| **19** | SS | FL | - | SS | 71 | 251299.9526 | 29 | -126043.371 | 29 |
| **20** | SS | SS | - | SS | 115 | 247667.0202 | 17 | -124470.6987 | 18 |
| **21** | FL | FL | FL | FL | 15 | 270559.8679 | 34 | -135363.0455 | 33 |
| **22** | FL | SS | FL | FL | 59 | 245060.7423 | 14 | -122857.2766 | 13 |
| **23** | SS | FL | FL | FL | 37 | 247087.3565 | 16 | -123748.6868 | 16 |
| **24** | SS | SS | FL | FL | 81 | 245586.3424 | 15 | -123241.9736 | 14 |
| **25** | FL | FL | SS | FL | 235 | 248624.1195 | 19 | -125614.1409 | 28 |
| **26** | FL | SS | SS | FL | 279 | 242284.7396 | 8 | -122688.2448 | 11 |
| **27** | SS | FL | SS | FL | 257 | 244051.9176 | 13 | -123449.9369 | 15 |
| **28** | SS | SS | SS | FL | 301 | 241561.6826 | 7 | -122448.6133 | 10 |
| **29** | FL | FL | FL | SS | 59 | 254674.3444 | 32 | -127664.0777 | 32 |
| **30** | FL | SS | FL | SS | 103 | 249889.7893 | 23 | -125515.594 | 26 |
| **31** | SS | FL | FL | SS | 81 | 253912.5738 | 31 | -127405.0893 | 31 |
| **32** | SS | SS | FL | SS | 125 | 249701.3153 | 21 | -125543.254 | 27 |
| **33** | FL | FL | SS | SS | 279 | 242399.9543 | 10 | -122745.8522 | 12 |
| **34** | FL | SS | SS | SS | 323 | 237318.7151 | 2 | -120449.0265 | 3 |
| **35** | SS | FL | SS | SS | 301 | 239960.4044 | 5 | -121647.9742 | 5 |
| **36** | SS | SS | SS | SS | 345 | 236303.5663 | 1 | -120063.349 | 1 |

Table comparing model fits using AIC and BIC 36 models were run within which the four types of model predictor in Eqn (4) (constant, size, environment, basal area) were left out or included with forest-level (FL) or species specific (SS) effects. Total number of parameters, AIC and BIC scores and rankings are reported. Models without size and species effects were rejected very strongly, and the additional inclusion of environmental and competition variables increased model fit significantly. The best-fit model, number 26, showed a very significant improvement on the next best using both AIC and BIC.
