# Supplementary material for: Influences of Forest Structure, Climate and Species Composition on Tree Mortality across the Eastern US
Source: PLoS One. 2010 Oct 13;5(10):e13212. doi: 10.1371/journal.pone.0013212 (PMC2954149; doi:10.1371/journal.pone.0013212)
Supplement: Table S2 — Table of maximum likelihood estimators (MLEs), Bayesian means and 2.5% and 97.5% confidence levels calculated from the posterior distributions for each of the 15 parameters of Eqn (9) for each of the 21 common species parameterised by the adaptive MCMC algorithm. The burn-in for the algorithm was 750,000 iterations and the sampling was 250,000 iterations. (0.18 MB DOC) [file pone.0013212.s002.doc]

**Table S2. Fitted parameter values for each of the 21 common species.**

Table S2

| **Parameter** | | **Species** |  |  |  |  |  |  |  |  |  |  |  |  |  |  |  |  |  |  |  |  |
| --- | --- | --- | --- | --- | --- | --- | --- | --- | --- | --- | --- | --- | --- | --- | --- | --- | --- | --- | --- | --- | --- | --- |
| (from Eqn (9)) | | *Acer rubrum* | *Pinus taeda* | *Quercus alba* | *Liquidambar styraciflua* | *Populus tremuloides* | *Acer saccarum* | *Quercus rubrum* | *Pinus echinata* | *Liriodendron tulipifera* | *Quercus velutina* | *Quercus prinus* | *Quercus stellata* | *Carya* spp | *Thuja occidentalis* | *Nyssa slyvatica* | *Quercus nigra* | *Betula papyferia* | *Fagus gra-ndifolia* | *Pinus virginiana* | *Fraxinus americana* | *N. sylv. (biflora)* |
| α_j_ | Bayesian Mean | -3.187 | -3.258 | -3.256 | -3.510 | -5.675 | -5.374 | -3.538 | -2.151 | -2.994 | -3.370 | -2.838 | -3.810 | -2.731 | -2.313 | -1.502 | -2.570 | -6.793 | -3.634 | -2.948 | -4.487 | -2.881 |
|  | MLE | -3.290 | -3.261 | -3.251 | -3.857 | -5.580 | -5.012 | -3.568 | -2.164 | -3.352 | -3.389 | -3.166 | -3.410 | -1.994 | -2.257 | -1.636 | -2.496 | -6.313 | -2.650 | -2.865 | -4.509 | -2.730 |
|  | 2.5% | -3.613 | -3.540 | -3.631 | -4.067 | -5.960 | -5.830 | -3.932 | -2.647 | -3.896 | -3.742 | -3.447 | -4.162 | -3.185 | -3.108 | -2.502 | -3.279 | -7.546 | -5.030 | -3.700 | -5.073 | -3.474 |
|  | 97.5% | -2.849 | -2.953 | -2.952 | -2.907 | -5.470 | -4.887 | -3.198 | -1.732 | -2.237 | -3.014 | -2.114 | -3.402 | -2.211 | -1.626 | -0.621 | -1.805 | -6.233 | -2.139 | -2.245 | -3.858 | -2.204 |
| β_1j_ | Bayesian Mean | -0.279 | -0.362 | -0.308 | -0.254 | -0.131 | -0.319 | -0.214 | -0.344 | -0.290 | -0.207 | -0.390 | -0.333 | -0.316 | -0.226 | -0.265 | -0.227 | -0.103 | -0.363 | -0.502 | -0.251 | -0.276 |
|  | MLE | -0.284 | -0.366 | -0.305 | -0.265 | -0.135 | -0.320 | -0.214 | -0.347 | -0.275 | -0.215 | -0.388 | -0.335 | -0.304 | -0.266 | -0.247 | -0.231 | -0.125 | -0.359 | -0.497 | -0.235 | -0.263 |
|  | 2.5% | -0.296 | -0.376 | -0.324 | -0.271 | -0.146 | -0.346 | -0.233 | -0.368 | -0.319 | -0.223 | -0.421 | -0.354 | -0.343 | -0.277 | -0.308 | -0.248 | -0.138 | -0.408 | -0.548 | -0.279 | -0.314 |
|  | 97.5% | -0.261 | -0.349 | -0.293 | -0.238 | -0.116 | -0.293 | -0.193 | -0.319 | -0.264 | -0.190 | -0.359 | -0.313 | -0.291 | -0.173 | -0.224 | -0.206 | -0.070 | -0.315 | -0.454 | -0.223 | -0.238 |
| β_2j_ | Bayesian Mean | -0.045 | -0.030 | -0.028 | -0.032 | -0.047 | -0.039 | -0.032 | -0.029 | -0.026 | -0.030 | -0.030 | -0.033 | -0.036 | -0.055 | -0.035 | -0.033 | -0.053 | -0.040 | -0.040 | -0.030 | -0.037 |
|  | MLE | -0.045 | -0.030 | -0.028 | -0.032 | -0.047 | -0.040 | -0.033 | -0.029 | -0.027 | -0.030 | -0.029 | -0.033 | -0.036 | -0.056 | -0.035 | -0.033 | -0.056 | -0.041 | -0.039 | -0.030 | -0.038 |
|  | 2.5% | -0.047 | -0.031 | -0.029 | -0.033 | -0.050 | -0.041 | -0.034 | -0.031 | -0.027 | -0.032 | -0.031 | -0.034 | -0.038 | -0.060 | -0.037 | -0.034 | -0.061 | -0.043 | -0.042 | -0.032 | -0.040 |
|  | 97.5% | -0.043 | -0.029 | -0.027 | -0.031 | -0.045 | -0.037 | -0.031 | -0.028 | -0.024 | -0.029 | -0.029 | -0.032 | -0.034 | -0.050 | -0.032 | -0.031 | -0.046 | -0.038 | -0.038 | -0.027 | -0.034 |
| β_3j_ | Bayesian Mean | 0.437 | -1.146 | 0.395 | -0.104 | 0.359 | 0.320 | 0.551 | -0.185 | -0.085 | 0.939 | 0.885 | -0.285 | 0.100 | 0.158 | -0.403 | -0.545 | 0.539 | 0.510 | -0.163 | 0.899 | 0.180 |
|  | MLE | 0.429 | -1.152 | 0.416 | -0.092 | 0.378 | 0.325 | 0.536 | -0.139 | -0.185 | 0.879 | 0.844 | -0.316 | 0.084 | 0.162 | -0.377 | -0.505 | 0.472 | 0.444 | -0.223 | 0.975 | 0.145 |
|  | 2.5% | 0.378 | -1.232 | 0.280 | -0.205 | 0.257 | 0.174 | 0.441 | -0.380 | -0.279 | 0.819 | 0.496 | -0.468 | -0.055 | -0.307 | -0.727 | -0.689 | 0.363 | 0.355 | -0.473 | 0.742 | -0.063 |
|  | 97.5% | 0.491 | -1.054 | 0.502 | -0.009 | 0.458 | 0.456 | 0.660 | 0.020 | 0.124 | 1.046 | 1.262 | -0.101 | 0.279 | 0.624 | -0.079 | -0.407 | 0.722 | 0.667 | 0.141 | 1.068 | 0.425 |
| β_4j_ | Bayesian Mean | -0.163 | 0.367 | -0.257 | 0.041 | 0.351 | -0.583 | -0.255 | -0.147 | -0.083 | -0.279 | 0.369 | -0.143 | 0.274 | 0.103 | 0.135 | 0.216 | 0.149 | -0.230 | 0.458 | -0.463 | -0.183 |
|  | MLE | -0.143 | 0.372 | -0.242 | 0.056 | 0.388 | -0.557 | -0.264 | -0.162 | -0.109 | -0.295 | 0.410 | -0.130 | 0.315 | 0.100 | 0.137 | 0.220 | 0.078 | -0.218 | 0.328 | -0.436 | -0.155 |
|  | 2.5% | -0.203 | 0.316 | -0.310 | -0.008 | 0.227 | -0.693 | -0.308 | -0.220 | -0.228 | -0.333 | 0.232 | -0.218 | 0.201 | -0.302 | -0.144 | 0.150 | 0.016 | -0.331 | 0.298 | -0.553 | -0.288 |
|  | 97.5% | -0.124 | 0.419 | -0.200 | 0.090 | 0.470 | -0.466 | -0.203 | -0.081 | 0.050 | -0.229 | 0.503 | -0.070 | 0.347 | 0.504 | 0.375 | 0.285 | 0.280 | -0.137 | 0.623 | -0.374 | -0.082 |
| β_5j_ | Bayesian Mean | -0.340 | 0.015 | -0.139 | 0.124 | -1.916 | -0.501 | -0.169 | -0.340 | 0.439 | -0.137 | 0.158 | -0.171 | 0.986 | -0.471 | -0.156 | -0.469 | -2.117 | 0.439 | -0.081 | 0.479 | 0.133 |
|  | MLE | -0.344 | -0.061 | -0.108 | 0.065 | -2.026 | -0.468 | -0.189 | -0.388 | 0.349 | -0.118 | 0.097 | -0.117 | 0.967 | -0.192 | -0.056 | -0.365 | -1.365 | 0.481 | -0.024 | 0.452 | 0.125 |
|  | 2.5% | -0.415 | -0.161 | -0.275 | -0.096 | -2.185 | -0.704 | -0.291 | -0.590 | 0.040 | -0.279 | -0.167 | -0.421 | 0.684 | -1.040 | -0.635 | -0.881 | -2.557 | 0.233 | -0.336 | 0.249 | -0.253 |
|  | 97.5% | -0.270 | 0.173 | 0.004 | 0.338 | -1.648 | -0.302 | -0.040 | -0.089 | 0.804 | -0.002 | 0.501 | 0.095 | 1.292 | 0.111 | 0.257 | -0.136 | -1.755 | 0.658 | 0.193 | 0.721 | 0.534 |
| β_6j_ | Bayesian Mean | 0.087 | -0.295 | 0.060 | -0.112 | -0.695 | -0.265 | 0.266 | 0.222 | -0.466 | 0.062 | -0.303 | -0.191 | -0.671 | -0.197 | 0.033 | -0.067 | -0.895 | -0.320 | -0.208 | -0.009 | -0.090 |
|  | MLE | 0.101 | -0.354 | 0.046 | -0.047 | -0.640 | -0.230 | 0.260 | 0.142 | -0.396 | 0.073 | -0.247 | -0.142 | -0.691 | -0.171 | 0.067 | -0.047 | -0.808 | -0.308 | -0.224 | 0.037 | 0.064 |
|  | 2.5% | 0.045 | -0.392 | -0.048 | -0.233 | -0.799 | -0.356 | 0.200 | 0.100 | -0.686 | -0.037 | -0.560 | -0.356 | -0.874 | -0.462 | -0.192 | -0.237 | -1.101 | -0.482 | -0.387 | -0.156 | -0.288 |
|  | 97.5% | 0.129 | -0.192 | 0.162 | 0.006 | -0.599 | -0.179 | 0.328 | 0.343 | -0.216 | 0.160 | -0.075 | -0.031 | -0.456 | 0.066 | 0.291 | 0.136 | -0.755 | -0.171 | -0.035 | 0.121 | 0.115 |
| β_7j_ | Bayesian Mean | 0.625 | 2.915 | -0.134 | 0.706 | -2.849 | 1.424 | 0.426 | -0.146 | 2.117 | -0.722 | 0.243 | 0.048 | 1.563 | 2.108 | -1.433 | -0.018 | -2.116 | -0.239 | 2.431 | -0.133 | 1.531 |
|  | MLE | 0.635 | 2.886 | -0.019 | 0.679 | -2.878 | 1.407 | 0.417 | -0.194 | 2.148 | -0.757 | -0.181 | -0.446 | 1.520 | 2.642 | -0.654 | 0.129 | -0.116 | -0.219 | 2.615 | -0.034 | 1.496 |
|  | 2.5% | 0.554 | 2.762 | -0.346 | 0.290 | -2.983 | 1.179 | 0.202 | -0.856 | 1.610 | -0.940 | -0.450 | -0.439 | 1.061 | 1.261 | -2.269 | -1.028 | -2.862 | -0.474 | 1.880 | -0.398 | 0.910 |
|  | 97.5% | 0.700 | 2.994 | 0.073 | 1.136 | -2.661 | 1.695 | 0.673 | 0.426 | 2.589 | -0.498 | 0.952 | 0.535 | 1.964 | 2.831 | -0.673 | 0.949 | -1.153 | -0.001 | 2.895 | 0.138 | 2.224 |
| β_8j_ | Bayesian Mean | 0.240 | -1.237 | -0.066 | -0.391 | -1.064 | 1.126 | -0.114 | -0.142 | -1.039 | -0.343 | -1.636 | -0.167 | -1.411 | 0.668 | 0.565 | -0.054 | -0.537 | 0.656 | -2.411 | 0.111 | -1.094 |
|  | MLE | 0.249 | -1.246 | -0.061 | -0.503 | -1.065 | 1.154 | -0.154 | -0.178 | -1.128 | -0.379 | -1.805 | -0.016 | -1.450 | 0.769 | 0.548 | 0.039 | -0.348 | 0.639 | -2.332 | 0.100 | -1.041 |
|  | 2.5% | 0.195 | -1.328 | -0.209 | -0.648 | -1.133 | 0.965 | -0.282 | -0.522 | -1.399 | -0.524 | -2.217 | -0.476 | -1.720 | 0.414 | 0.207 | -0.545 | -0.813 | 0.522 | -2.896 | -0.071 | -1.527 |
|  | 97.5% | 0.284 | -1.132 | 0.082 | -0.150 | -0.983 | 1.272 | 0.061 | 0.313 | -0.672 | -0.166 | -1.080 | 0.143 | -1.072 | 0.938 | 0.945 | 0.440 | -0.145 | 0.794 | -1.779 | 0.287 | -0.701 |
| β_9j_ | Bayesian Mean | -0.264 | -0.276 | -0.144 | 0.013 | 0.086 | 0.057 | -0.072 | -0.108 | 0.304 | -0.699 | -0.261 | -0.037 | 0.040 | 0.311 | -0.277 | -0.242 | 0.389 | -0.008 | 0.297 | 0.195 | -0.027 |
|  | MLE | -0.282 | -0.217 | -0.141 | -0.088 | 0.076 | 0.024 | -0.053 | -0.054 | 0.111 | -0.701 | -0.332 | -0.017 | 0.013 | 0.314 | -0.109 | -0.258 | 0.415 | -0.021 | 0.227 | 0.343 | -0.161 |
|  | 2.5% | -0.307 | -0.362 | -0.261 | -0.079 | 0.028 | -0.064 | -0.187 | -0.261 | 0.130 | -0.817 | -0.546 | -0.176 | -0.169 | 0.121 | -0.472 | -0.402 | 0.291 | -0.136 | 0.078 | 0.016 | -0.223 |
|  | 97.5% | -0.219 | -0.188 | -0.019 | 0.101 | 0.138 | 0.167 | 0.044 | 0.040 | 0.472 | -0.587 | 0.033 | 0.116 | 0.217 | 0.493 | -0.087 | -0.086 | 0.492 | 0.126 | 0.529 | 0.375 | 0.167 |
| β_10j_ | Bayesian Mean | 0.100 | -0.417 | 0.033 | 0.022 | 0.003 | 0.058 | 0.064 | -0.085 | 0.262 | 0.190 | 0.719 | 0.035 | 0.179 | 0.013 | -0.107 | -0.058 | 0.107 | 0.210 | 0.961 | 0.148 | -0.008 |
|  | MLE | 0.104 | -0.407 | 0.046 | -0.019 | 0.004 | 0.048 | 0.060 | -0.082 | 0.277 | 0.171 | 0.655 | 0.003 | 0.190 | 0.016 | -0.087 | -0.075 | 0.060 | 0.161 | 1.006 | 0.137 | -0.005 |
|  | 2.5% | 0.053 | -0.490 | -0.016 | -0.041 | -0.028 | -0.002 | 0.020 | -0.156 | 0.153 | 0.143 | 0.507 | -0.026 | 0.074 | -0.138 | -0.243 | -0.154 | 0.054 | 0.087 | 0.779 | 0.075 | -0.108 |
|  | 97.5% | 0.143 | -0.344 | 0.080 | 0.086 | 0.034 | 0.117 | 0.107 | -0.012 | 0.377 | 0.243 | 0.927 | 0.095 | 0.280 | 0.164 | 0.028 | 0.035 | 0.156 | 0.328 | 1.151 | 0.218 | 0.094 |
| β_11j_ | Bayesian Mean | 0.389 | 0.336 | 0.239 | -0.209 | 0.295 | -1.650 | 0.491 | 0.556 | 0.159 | 0.313 | 1.105 | -0.527 | 0.866 | -0.190 | 0.036 | 0.206 | -0.246 | -0.615 | 0.131 | -1.220 | 0.419 |
|  | MLE | 0.291 | 0.381 | 0.165 | -0.262 | 0.157 | -1.701 | 0.547 | 0.535 | 0.153 | 0.332 | 1.142 | -0.318 | 1.067 | -0.014 | -0.079 | 0.345 | -0.109 | 0.000 | -0.082 | -0.557 | 0.009 |
|  | 2.5% | 0.159 | 0.188 | -0.088 | -0.538 | 0.111 | -2.056 | 0.270 | 0.216 | -0.727 | 0.071 | 0.681 | -0.983 | 0.583 | -0.416 | -0.633 | -0.142 | -0.450 | -1.691 | -0.579 | -1.627 | 0.072 |
|  | 97.5% | 0.588 | 0.539 | 0.488 | 0.227 | 0.458 | -1.335 | 0.710 | 0.918 | 0.818 | 0.703 | 1.418 | -0.239 | 1.105 | 0.060 | 0.489 | 0.610 | -0.038 | 0.694 | 0.675 | -0.680 | 0.730 |
| β_12j_ | Bayesian Mean | 0.129 | 0.092 | 0.108 | 0.040 | 0.104 | -0.294 | 0.128 | 0.231 | 0.117 | 0.048 | 0.388 | -0.088 | 0.111 | -0.012 | 0.003 | 0.080 | 0.002 | -0.133 | -0.006 | -0.175 | 0.124 |
|  | MLE | 0.110 | 0.098 | 0.113 | 0.035 | 0.075 | -0.134 | 0.147 | 0.250 | 0.068 | 0.109 | 0.535 | -0.007 | 0.157 | -0.028 | 0.000 | 0.085 | 0.014 | -0.035 | -0.024 | -0.109 | 0.053 |
|  | 2.5% | 0.088 | 0.060 | 0.017 | -0.016 | 0.071 | -0.378 | 0.062 | 0.110 | -0.044 | -0.042 | 0.201 | -0.228 | 0.007 | -0.051 | -0.110 | 0.014 | -0.036 | -0.366 | -0.295 | -0.271 | 0.049 |
|  | 97.5% | 0.165 | 0.133 | 0.178 | 0.119 | 0.135 | -0.216 | 0.193 | 0.337 | 0.237 | 0.177 | 0.529 | 0.007 | 0.202 | 0.029 | 0.081 | 0.156 | 0.041 | 0.153 | 0.232 | -0.058 | 0.191 |
| β_13j_ | Bayesian Mean | 0.006 | 0.073 | 0.093 | 0.013 | 0.014 | 0.010 | 0.101 | 0.113 | 0.024 | 0.124 | 0.111 | 0.142 | 0.100 | -0.025 | -0.012 | 0.047 | 0.012 | -0.018 | 0.131 | -0.002 | 0.017 |
|  | MLE | 0.004 | 0.071 | 0.035 | 0.017 | 0.015 | 0.022 | 0.102 | 0.112 | -0.015 | 0.106 | 0.029 | 0.130 | 0.031 | -0.020 | -0.006 | 0.032 | 0.007 | -0.008 | 0.120 | 0.001 | -0.004 |
|  | 2.5% | -0.003 | 0.062 | 0.068 | 0.001 | 0.006 | -0.006 | 0.082 | 0.089 | 0.003 | 0.106 | 0.075 | 0.120 | 0.071 | -0.037 | -0.025 | 0.017 | -0.001 | -0.030 | 0.092 | -0.014 | -0.010 |
|  | 97.5% | 0.018 | 0.085 | 0.115 | 0.025 | 0.022 | 0.030 | 0.123 | 0.137 | 0.055 | 0.145 | 0.143 | 0.168 | 0.125 | -0.007 | 0.002 | 0.067 | 0.026 | -0.004 | 0.176 | 0.012 | 0.050 |
| β_14j_ | Bayesian Mean | -2.0E-04 | -7.0E-04 | -1.6E-03 | -1.2E-04 | -2.4E-04 | -3.2E-04 | -2.1E-03 | -1.5E-03 | -6.0E-04 | -2.2E-03 | -1.9E-03 | -2.1E-03 | -1.8E-03 | 2.1E-04 | 9.8E-05 | -4.8E-04 | -2.6E-04 | -3.8E-05 | -2.1E-03 | -1.1E-04 | -5.2E-04 |
|  | MLE | 7.0E-06 | -7.4E-04 | -1.0E-03 | 1.0E-06 | -2.1E-04 | -1.7E-04 | -2.1E-03 | -1.5E-03 | -5.5E-04 | -1.9E-03 | -1.1E-03 | -6.6E-04 | -1.7E-03 | 2.2E-04 | 1.0E-04 | -4.2E-04 | -1.1E-04 | 5.1E-05 | -1.8E-03 | 0.0E+00 | -3.7E-04 |
|  | 2.5% | -3.6E-04 | -8.8E-04 | -1.9E-03 | -2.9E-04 | -3.8E-04 | -6.3E-04 | -2.5E-03 | -1.9E-03 | -1.1E-03 | -2.6E-03 | -2.4E-03 | -2.6E-03 | -2.3E-03 | -1.8E-05 | -5.1E-05 | -8.5E-04 | -4.8E-04 | -2.3E-04 | -2.8E-03 | -2.9E-04 | -1.0E-03 |
|  | 97.5% | -9.1E-05 | -5.4E-04 | -1.2E-03 | 5.1E-05 | -1.0E-04 | -8.7E-05 | -1.7E-03 | -1.1E-03 | -2.9E-04 | -1.9E-03 | -1.4E-03 | -1.7E-03 | -1.3E-03 | 3.7E-04 | 2.3E-04 | -4.5E-06 | -5.1E-05 | 7.5E-05 | -1.5E-03 | 1.3E-05 | -8.8E-05 |

Table of maximum likelihood estimators (MLEs), Bayesian means and 2.5% and 97.5% confidence levels calculated from the posterior distributions for each of the 15 parameters of Eqn (9) for each of the 21 common species parameterised by the adaptive MCMC algorithm. The burn-in for the algorithm was 750,000 iterations and the sampling was 250,000 iterations.
