# Supplementary figures and images for: Influences of Forest Structure, Climate and Species Composition on Tree Mortality across the Eastern US
Source: PLoS One. 2010 Oct 13;5(10):e13212. doi: 10.1371/journal.pone.0013212 (PMC2954149; doi:10.1371/journal.pone.0013212)

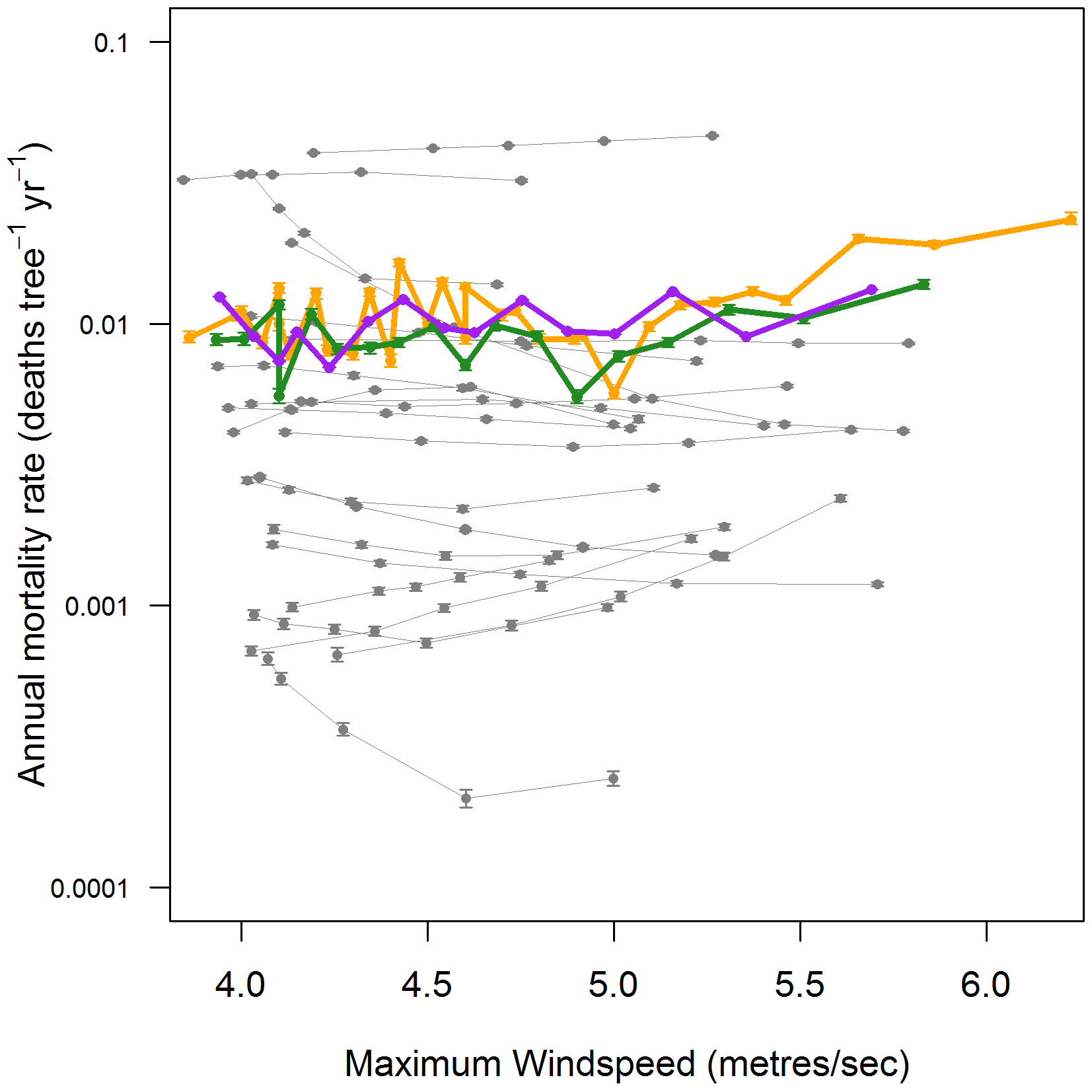

Supplement: Figure S1 — Observed and predicted mortality rates against maximum wind speed. Log annual mortality rates observed for the whole forest including rare species (orange) and the 21 common species (green), and the model predictions for the 21 species combined (purple) and each species individually (grey), plotted against maximum wind speed (m/sec). Species' error bars (grey) show parameter uncertainty, forest error bars (purple, orange and green) show the 95% confidence interval for the mortality rates predicted from the model-created and real datasets. (8.20 MB TIF) [file pone.0013212.s003.tif]

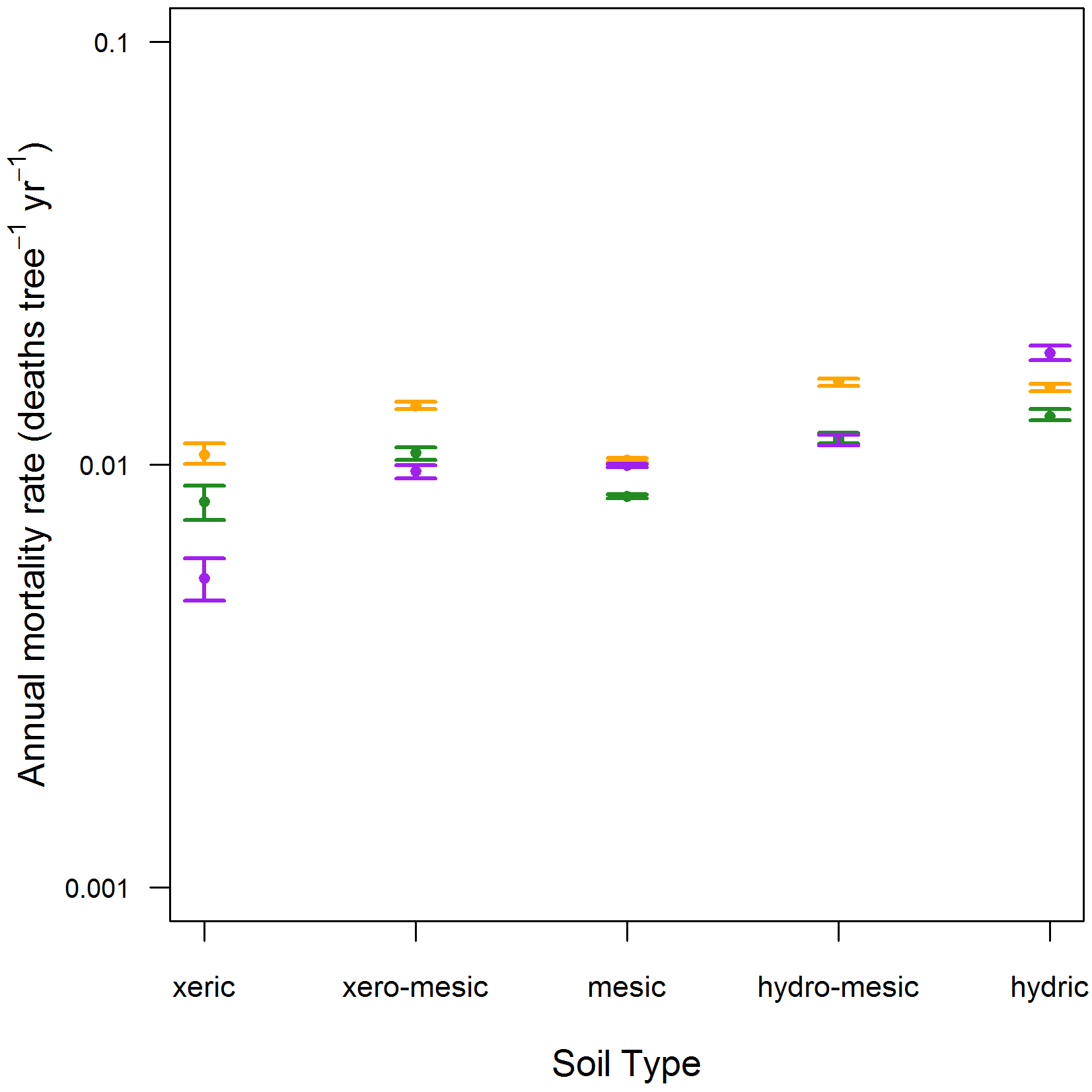

Supplement: Figure S2 — Observed and predicted mortality rates against soil type. Log annual mortality rates plotted against soil type for the predicted forest-level mortality rate for all 21 species parameterised by the model (purple), the real forest-level mortality rates for the 21 species (green) and the whole forest including rare species (orange). Error bars (purple, orange and green) show the 95% confidence interval for the mortality rates predicted from the model-created and real datasets. (8.20 MB TIF) [file pone.0013212.s004.tif]

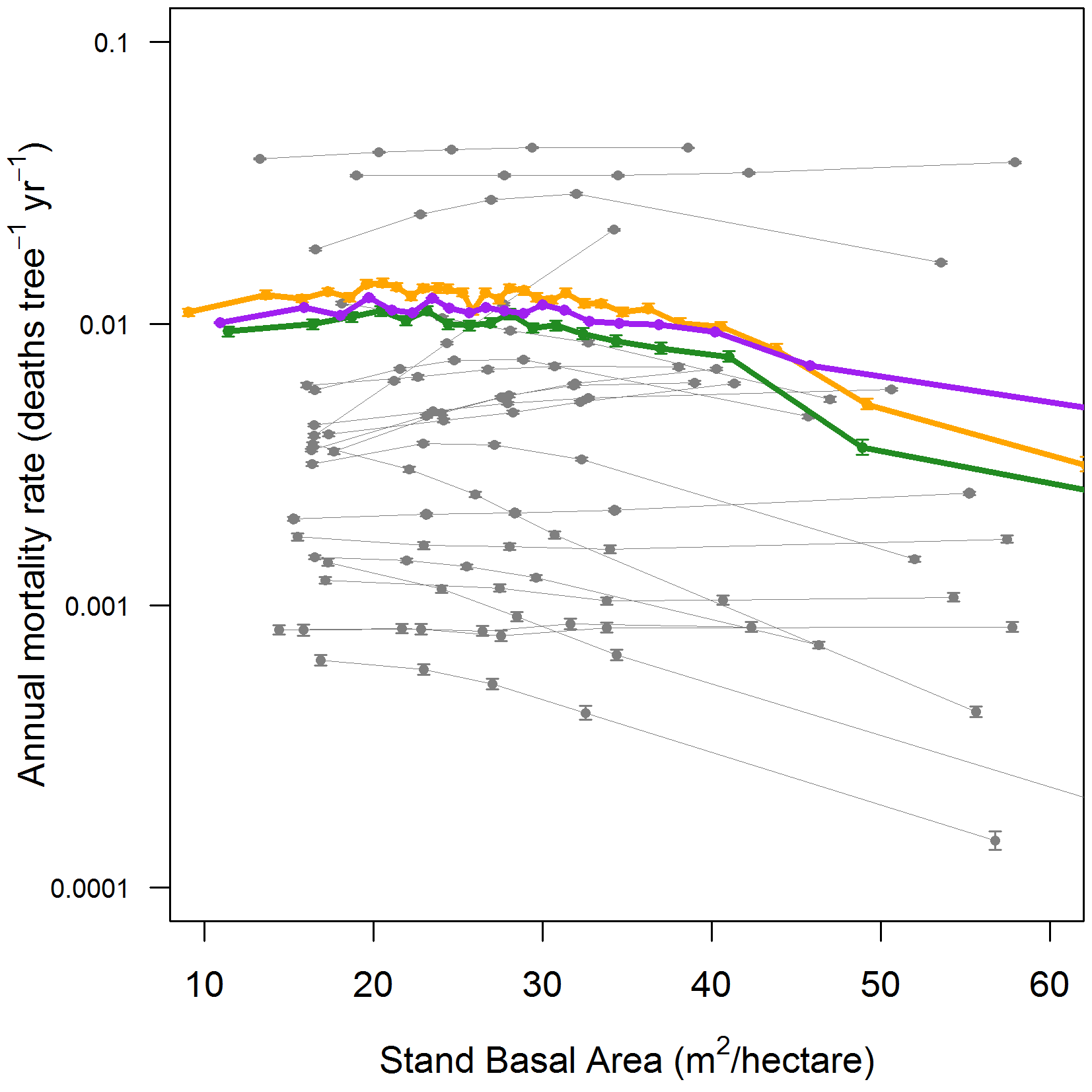

Supplement: Figure S3 — Observed and predicted mortality rates against plot basal area. Log annual mortality rates observed for the whole forest including rare species (orange) and the 21 common species (green), and the model predictions for the 21 species combined (purple) and each species individually (grey), plotted against plot basal area (m2/hectare). Species' error bars (grey) show parameter uncertainty, forest error bars (purple, orange and green) show the 95% confidence interval for the mortality rates predicted from the model-created and real datasets. (8.20 MB TIF) [file pone.0013212.s005.tif]

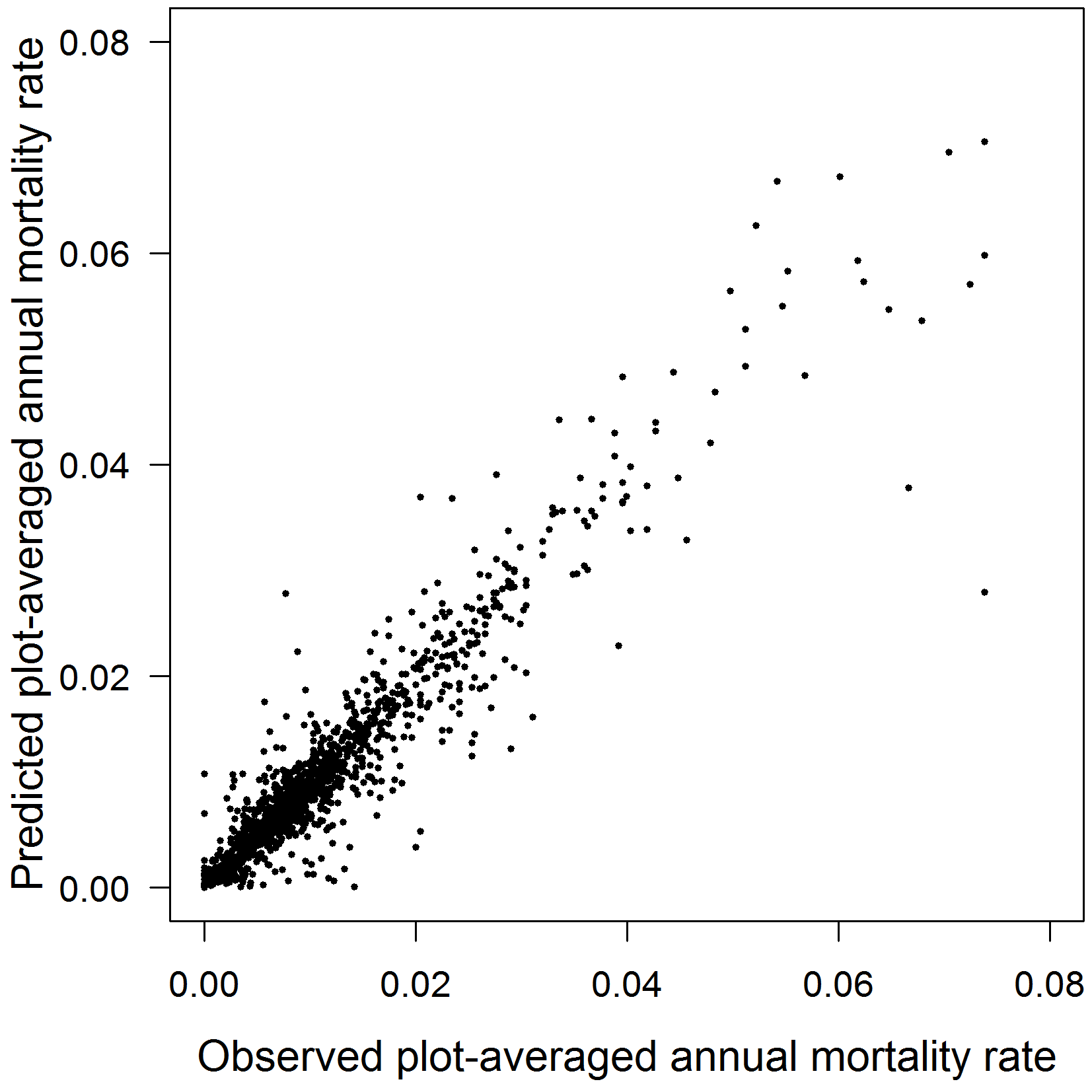

Supplement: Figure S4 — Observed versus predicted plot-averaged mortality rates. Observed versus predicted plot-averaged annual mortality rate for all plots with at least 10 stems, showing the high correlation (r2 = 0.9). (8.20 MB TIF) [file pone.0013212.s006.tif]

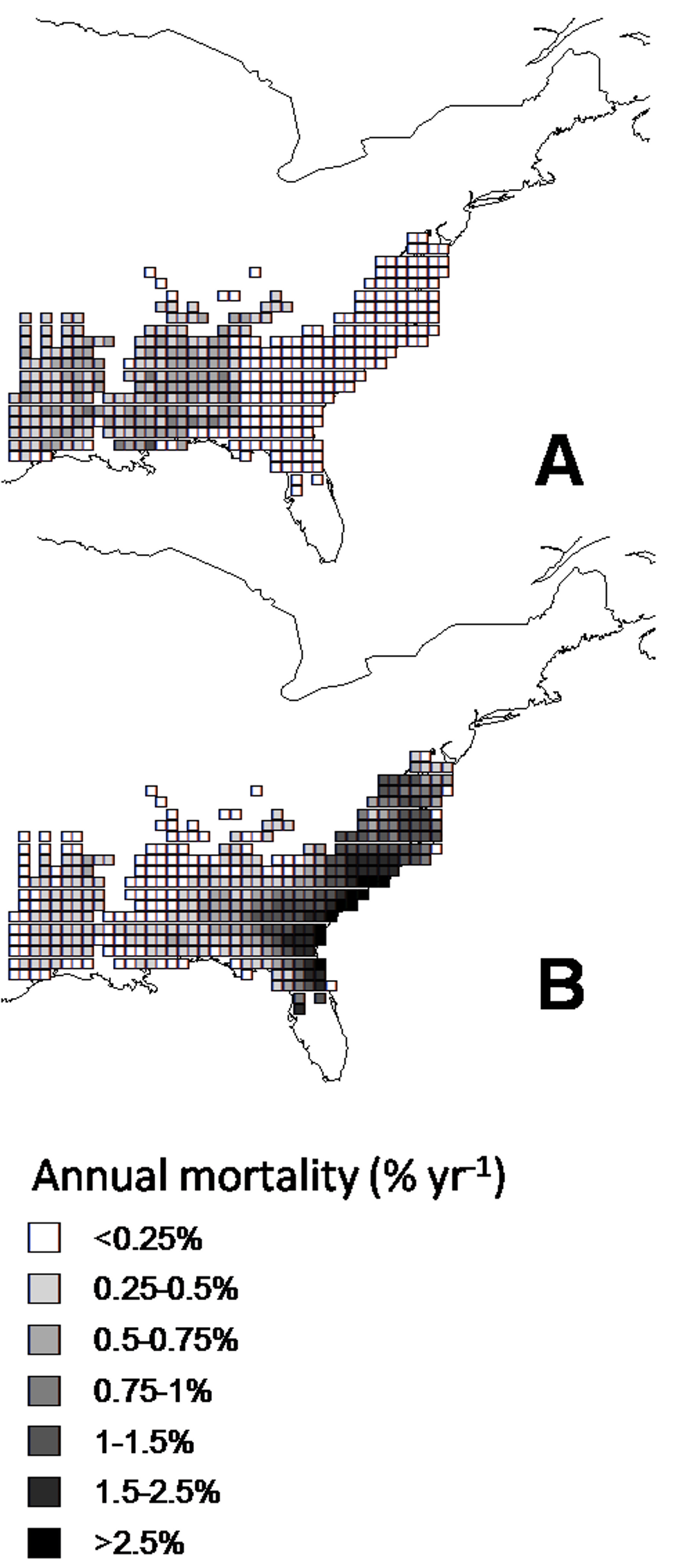

Supplement: Figure S5 — Patterns of mortality due to regional variation in stand strucuture and environmental alone. Maps of estimated annual forest-level mortality across the Eastern United States illustrating the contributions of variation in stand structure (stem size and plot basal area) and environment, modelled across the range of Pinus taeda to control for the effects of species composition. (A) Variation in forest structure alone (stem size and plot basal area), illustrated by removing environmental effects and modelling just the most common species (P. taeda). (B) The effect of variation in environment alone, illustrated by modelling P. taeda without stand structure variation (i.e. modelling a 20 cm dbh tree) across the region. (8.88 MB TIF) [file pone.0013212.s007.tif]

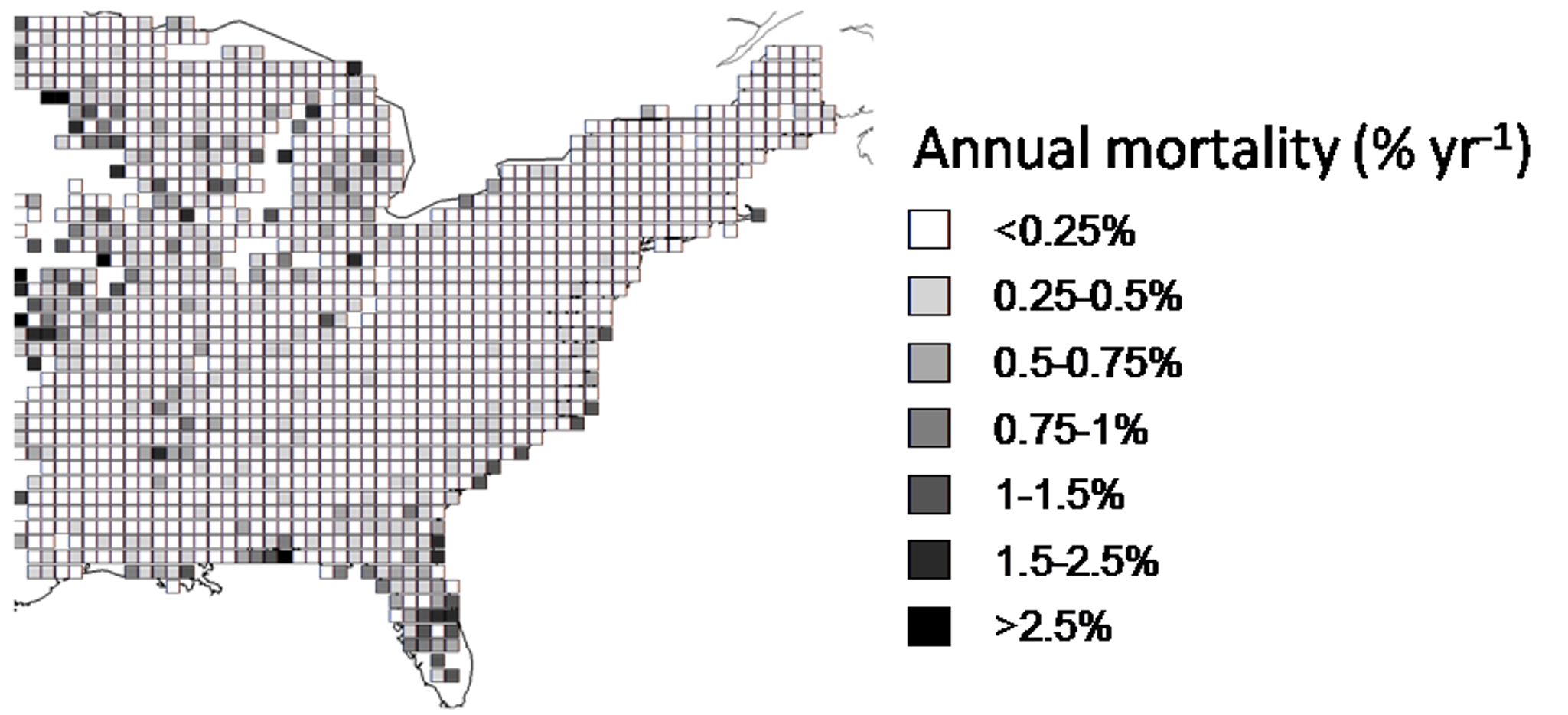

Supplement: Figure S6 — Regional patterns of differences between observed at predicted mortality rates. Map of absolute difference between predicted and observed forest level mortality across the Eastern United States. (7.66 MB TIF) [file pone.0013212.s008.tif]
